# Unveiling key descriptors *via* machine learning: toward rational molecular design of chromophores with excited-state intramolecular proton transfer

**DOI:** 10.1039/d5sc07051a

**Published:** 2026-02-09

**Authors:** Shengsheng Wei, Zipeng Yang, Chao Yang, Hongmei Zhao, Yang Li, Yuanyuan Guo, Andong Xia, Zhuoran Kuang

**Affiliations:** a State Key Laboratory of Information Photonic and Optical Communications, School of Physical Science and Technology, Beijing University of Posts and Telecommunications (BUPT) Beijing 100876 P.R China andongxia@bupt.edu.cn kuang@bupt.edu.cn

## Abstract

Precise design of excited-state intramolecular proton transfer (ESIPT) molecules targeting advanced optoelectronic or biological sensing applications presents a fundamental challenge. Controlling the energy difference (ΔE*) between normal (N*) and tautomeric (T*) excited-state forms is crucial, yet the complex interplay of hydrogen bond (H-bond) strength, proton donor acidity, and proton acceptor basicity with ΔE* remains insufficiently explored. Conventional trial-and-error approaches for designing tailored ESIPT compounds suffer from inefficient synthesis. To address this, we constructed a high-quality ESIPT dataset by introducing ten substituents with progressively increasing electron-donating capacity into six representative ESIPT parent scaffolds. Integrating qualitative descriptors with data-driven machine learning (ML) enabled precise ΔE* prediction, significantly accelerating high-throughput screening. An interpretable Shapley additive explanations (SHAP)-based ML approach was applied to evaluate the relative importance of key H-bond descriptors while achieving accurate ΔE* prediction. Novel ESIPT candidates were generated using a variational autoencoder (VAE) model and filtered using predicted ΔE*, synthetic accessibility (SA) scores, and pharmacokinetic properties. Critically, we synthesized two AI-designed ESIPT molecules exhibiting distinct N*/T* dual emission, which provides a closed-loop experimental validation of this data-driven molecular design strategy. This work establishes a predictive framework for accurate ΔE* determination and accelerated exploitation of novel promising ESIPT compounds.

## Abstract 2

This work introduces a data-driven, closed-loop strategy for ESIPT molecular design with ΔE* prediction, interpretation, and candidate generation. The strategy was validated by synthesizing two AI-designed molecules with distinct dual emission.

## Graphical abstract

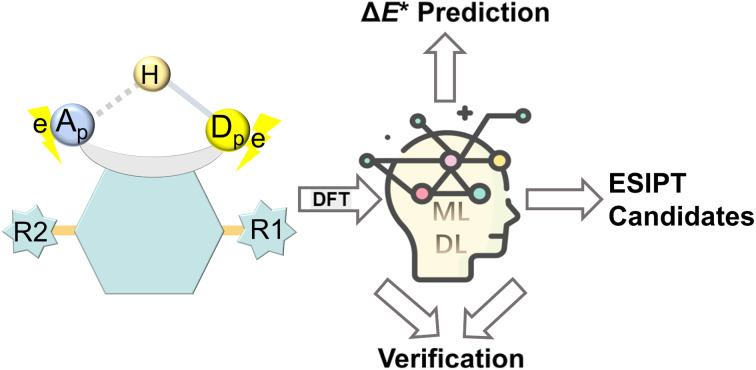

## Introduction

Excited-state intramolecular proton transfer (ESIPT) is a photophysical process wherein a photoexcited molecule undergoes proton-transfer isomerization from its excited normal (N*) to tautomeric (T*) configurations. ESIPT emitters have garnered substantial research interest owing to their exceptionally large Stokes-shifted emission, arising from the energy difference between N* and T* state (ΔE*), and pronounced microenvironment sensitivity to pH, solvent polarity, and viscosity. These properties render ESIPT-based materials highly promising for bioimaging probes with ratiometric detection capability,^1–5^ spectrum-tunable organic light-emitting diodes (OLEDs), and single-molecule white-light emitters.^6–13^ Numerous studies have focused on modulating ΔE* to control the ESIPT kinetics reaction. Molecular engineering with strategic modification of electron-donating groups (EDGs) or electron-withdrawing groups (EWGs) and microenvironment tuning of media polarization or viscosity modulate ΔE* in experiments.^14–16^ Computational methods, such as time-dependent density functional theory (TD-DFT) or complete active space self-consistent field (CASSCF), enable ΔE* determination *via* N* and T* energy calculations, thereby circumventing the high experimental costs.^15,17^ However, the computational burden escalates with molecular size and dataset scale, limiting its applicability in high-throughput screening.

Artificial intelligence (AI) has revolutionized high-throughput molecular screening by significantly accelerating the discovery of promising candidates. Central to this advancement are interpretable property prediction models, AI systems that predict molecular properties while decoding structural–activity relationship. The integration of interpretable machine learning (ML) methods has shifted the paradigm from empirical optimization or purely data-driven approaches to mechanism-oriented discovery frameworks, quantitatively resolving feature importance to extract chemical design principles. ML-based property-prediction models have enabled high-throughput screening across diverse functional materials, including thermally activated delayed fluorescence (TADF) emitters,^18,19^ lithium battery electrolytes,^20,21^ solid-state optical materials,^22^ organic photovoltaics,^23,24^ nonlinear optical crystals,^25,26^*etc.* However, despite these successes, the reliance of property-prediction models on pre-enumerated molecular libraries can become a bottleneck when aiming to explore ultra-large or uncharted regions of chemical space within a practical timeframe.^27–29^

**Fig. 1:**
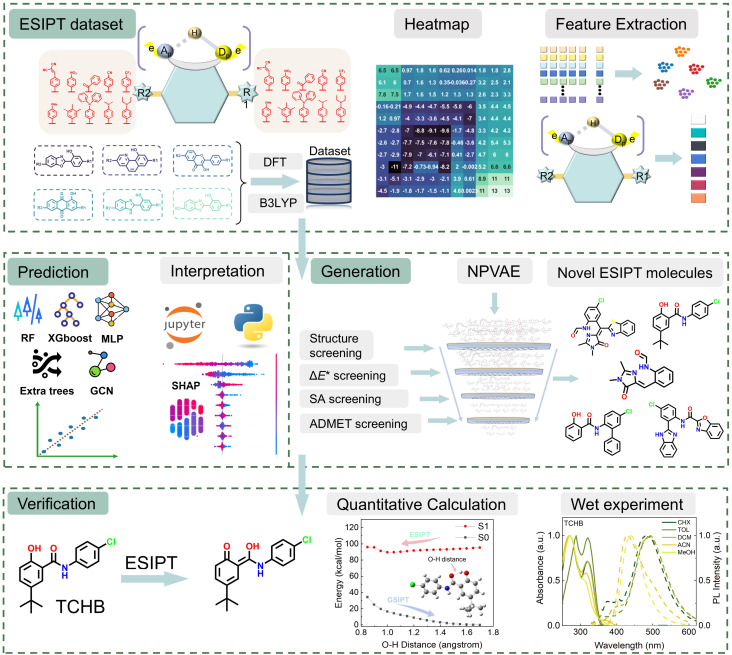
Schematic representation of identifying promising ESIPT molecules utilizing AI, quantitative computational analysis, and experimental verification.

In contrast to the property-prediction model, generative AI enables *de novo* design of molecular structures with target properties while bypassing costly enumeration-evaluation cycles. These models explore expansive chemical spaces beyond predefined *in silico* libraries, facilitating the discovery of structurally innovative motifs critical for breakthrough applications.^30–36^ However, a fundamental limitation persists in that a large proportion of AI-generated compounds exhibit synthetic inaccessibility due to unrealistic ring strain, forbidden bond angles, or lack of retrosynthetic pathways, rendering experimental validation unfeasible. Consequently, there has been growing interest in developing generative AI models that can design synthesizable molecules. Although several methods have shown promising *in silico* results, very few studies undergo experimental synthesis validation.^18,20,36–39^ Moreover, only a few studies have attempted ΔE* prediction and ESIPT molecular design using AI methods, such as the work by Zeng *et al*.^27^ and Raucci.^40^ Nevertheless, the extensive literature on ΔE* modulation provides valuable insights, inspiring our investigation into the key descriptors governing ESIPT behaviour.^14–17,41–46^ In this study, we presented an integrated framework combining quantum-chemical calculations, ML, and experimental validation to discover novel ESIPT molecules (Fig. 1). A high-quality ESIPT dataset was constructed through theoretical calculations and analyzed *via* statistical methods and substituent-ΔE* heatmaps. Molecular descriptors generated using RDKit and DeepChem^47,48^ were visualized by t-distributed stochastic neighbor embedding (t-SNE)^49^ and principal component analysis (PCA). Subsequent ML model training enabled accurate prediction of ΔE*. Employing an interpretable algorithm, Shapley additive explanations (SHAP),^50^ we identified and rationalized key descriptors influencing ΔE* (*e.g.*, hydrogen-bond length difference between N* and N) and evaluated their relative importance. To explore novel chemical space, we utilized a variational autoencoder (NPVAE)^36,51,52^ to generate promising ESIPT candidates. These candidates were rigorously filtered based on predicted ΔE*, ADMET (absorption, distribution, metabolism, excretion, and toxicity) properties, and synthetic accessibility (SA) scores. Notably, two prioritized candidates synthesized for experimental validation exhibited distinct N* and T* emissions, validating our data-driven molecular design strategy. This work demonstrates the power of integrating ML, TD-DFT calculations, and experiments for the efficient design of functional ESIPT systems.

## Results and discussion

### Construction of the ESIPT dataset

A high-quality ESIPT dataset containing ΔE* was systematically constructed to enable accurate ΔE* prediction and AI-based molecular design. Initial ESIPT-active molecules were collected through comprehensive literature screening. From this collection, eighteen parent ESIPT molecular scaffolds were extracted, where ΔE* was calculated from eleven of them (Table S1). These scaffolds were classified into five categories based on proton donor-proton acceptor interactions: OH⋯O, OH⋯N, NH⋯N, N(R)H⋯N, and NH⋯O (Fig. S1). Six representative scaffolds were selected for dataset construction: 2-(2′-hydroxyphenyl)benzoxazole (HBO), 10-hydroxybenzo[*h*]quinoline (HBQ), 3-hydroxyflavone (3HF), 1-hydroxyanthraquinones (HAQ), 2-(2′-hydroxyphenyl)benzimidazole (HBI), and 2-(2′-hydroxyphenyl)benzothiazole (HBT). Selection criteria included: (1) intrinsic ultrafast ESIPT kinetics and (2) extensive literature validation of their stability as ESIPT-active compounds. All six scaffolds feature proton donor/acceptor moieties integrated within five- or six-membered ring systems.

**Fig. 2:**
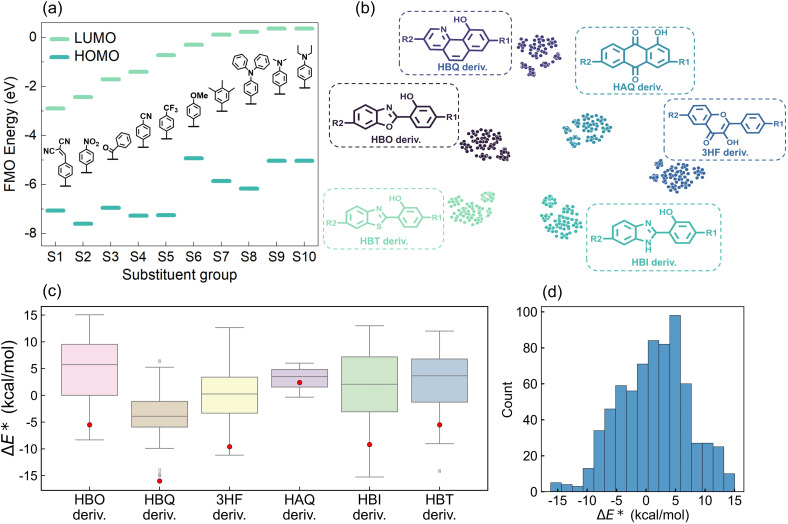
(a) Frontier molecular orbital (FMO) energy level alignment of substituent groups. (b) Chemical space visualization of the ESIPT dataset based on the t-SNE clustering method. Points represent individual molecules color-coded by derivative (deriv.) class: HBO, HBQ, HAQ, 3HF, HBI, and HBT. (c) Distributions of ΔE* for the six types of derivatives, respectively. The red dots represent the ΔE* values of the parent ESIPT molecules. (d) Distribution of ΔE* for the ESIPT molecular dataset.

To maximize chemical diversity, ten substituents, ranked by electron-withdrawing strength (from strongest to weakest) based on LUMO energies (Fig. 2a), were systematically introduced at R1 and R2 positions along the long molecular axis of each scaffold. Ground-state (N) and excited-state (N*, T*) geometries of all 704 derivatives underwent full geometric optimization using density functional theory (DFT) and time-dependent DFT (TD-DFT) with the B3LYP functional, ensuring convergence to energy minima. This yielded the final ΔE* dataset for quantitative analysis (see SI Files, XLSX file 1 for the ESIPT dataset). Data visualization provides critical insights for intuitive dataset interpretation. To map chemical space, molecules were clustered using ECFP^53^ and visualized *via* t-SNE. The t-SNE algorithm projects structural similarities into 2D space with proximate points indicating molecular resemblance. The ESIPT derivatives classified as HBO, HBQ, 3HF, HAQ, HBI, and HBT types exhibit distinct clustering in the t-SNE plot (Fig. 2b), validating the classification approach. The clear classification of the six groups not only demonstrates the effectiveness of the clustering method and the fingerprint, but also indirectly confirms the capability of the subsequent AI algorithm to recognize molecular structures based on fingerprints. Systematic derivatization significantly modulates the ΔE* relative to parent molecules (Fig. 2c). For instance, HBI parent exhibits ΔE* = −9.2 kcal mol^−1^, while derivatization extends this range to approximately −15 to 13 kcal mol^−1^, indicating effective ESIPT thermodynamic tuning *via* excited-state intramolecular charge transfer (ESICT). Similar ΔE* modulation breadth was observed for five other derivative classes. Notably, HAQ derivatives show a narrow ΔE* distribution, suggesting skeletal vibration-dominated ESIPT rather than ESICT mediated processes.^54–56^ The calculated ΔE* of all compounds spans between −16 and 15 kcal mol^−1^ with an average of 1.32 kcal mol^−1^ (Fig. 2d).

To elucidate substituent effects on ESIPT energetics, ΔE* regulation heatmaps were generated for all six derivative classes (Fig. S2). Crucially, ΔE* variations exhibited no monotonic correlation with substituent electron-withdrawing strength (as ranked by LUMO energies), demonstrating that ESICT serves as merely one contributing factor in dictating ESIPT thermodynamics. The substituent-ΔE* heatmaps indicate that ΔE* values of modified derivatives are almost higher than those of their corresponding parent molecules. Using 3HF derivatives as an example, compared to the ΔE* of 3HF which is −9.6 kcal mol^−1^, most of the 3HF derivatives show a higher value, except when the R1 site is connected to the S3 substituent and the R2 site is attached to the S2 substituent, in which case the value is −11 kcal mol^−1^ (Fig. S2c). For other types of derivatives, the cases in which ΔE* is lower than that of the corresponding parent molecules are rarely observed. Additionally, substitution of EDGs (S8, S9, and S10) at R1 and R2 positions consistently resulted in higher ΔE* values, systematically exceeding those observed for EWG substitutions. Though ΔE* trends deviate from simple substituent FMO's energetic ordering, substituents regulate ESIPT through synergistic electronic and steric effects, necessitating multidimensional descriptors for predictive modeling and laying the groundwork for machine learning applications.

### ΔE* prediction using ML with multidimensional descriptors

To predict ΔE* using ML, molecules were characterized using three complementary descriptor categories: quantitative descriptors (209-dimensional features), qualitative descriptors (encode 2048-bit in length), and molecular graphs (represented by DMPNN feature^28^), which were computed by RDkit and DeepChem.^47,48^ This multifaceted approach effectively captures quantitative, qualitative, and structural properties of the ESIPT dataset. Ten popular ML algorithms were then employed for ΔE* prediction. Algorithm performance was comprehensively evaluated using the mean absolute error (MAE), root-mean-square error (RMSE), and the squared Pearson correlation coefficient (*R*^2^). In addition to ML algorithms, five graph convolution models, such as Attentive FP Model, GAT Model, *etc.*, were also applied to predict ΔE* with MAE reaching 2.91 kcal mol^−1^. In our experiment, 5-fold cross-validation (CV) was used to evaluate different algorithms in combination with various molecular descriptors and select hyperparameters (see SI Files, XLSX file 2 for the full list of 5-fold CV results and model configurations for ML algorithms and graph convolution models).

**Fig. 3:**
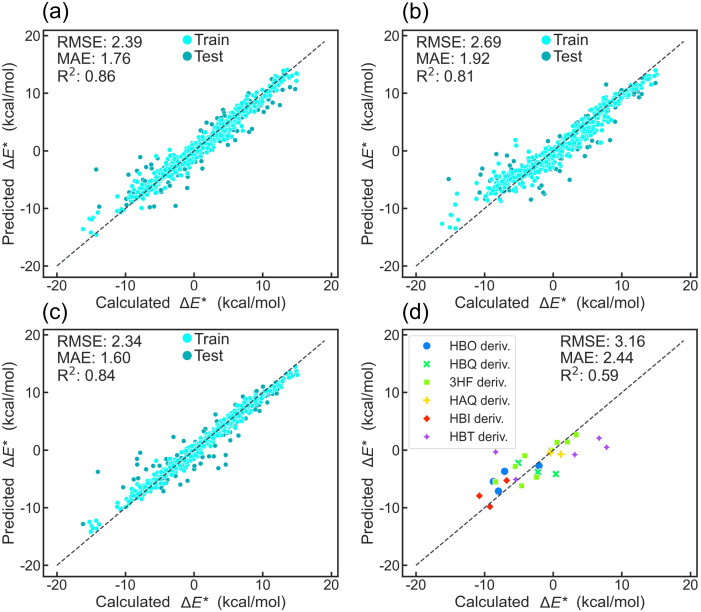
The plot of predicted *versus* calculated ΔE* of (a) GB, (b) RF, and (c) XGBoost models used atom-pair fingerprints as input at one of the folds in the ESIPT dataset. (d) The plot of predicted *versus* calculated values of the XGBoost model, which used atom-pair fingerprints as input for documented ESIPT molecules.

The atom-pair fingerprint^57^ consistently outperforms all fingerprints across all evaluated metrics (Fig. S3–S12). This superiority was further elucidated by t-SNE visualization, which reveals a distinct clustering pattern between atom-pair and other fingerprints (RDKit, ECFP, and topological torsion). While other fingerprints form six well-separated clusters based on parent molecule types, the atom-pair fingerprint exhibits a more complex and less clustered distribution (Fig. S14). Notably, HBO, HBI, and HBT derivatives appear as paired clusters, reflecting their shared structural features: an NX2-(5)-OX1 atom pair (Fig. S14e). The distinct distribution pattern of atom-pair fingerprints is also observed in the PCA visualization (Fig. S15). Among all ML algorithms, XGBoost, Random Forest (RF), and Gradient Boosting (GB) rank highest in predictive performance. The combination of the XGBoost model and atom-pair descriptors delivers the best results, achieving an average MAE of 1.55 kcal mol^−1^ through 5-fold CV. It outperforms RF and GB in terms of MAE at one of the folds, reaching 1.60 kcal mol^−1^ (Fig. 3a–c). Test data points closely align with the ideal prediction line, mirroring the training set distribution, indicating the robust ability of XGBoost to capture the atom-pair descriptors and ΔE* relationship. To assess the generalization capability of the model, the XGBoost–atom-pair combination was applied to predict ΔE* for documented ESIPT molecules (see SI Files, XLSX file 3). The model demonstrated reliable accuracy for molecules that share parent scaffolds with those in the training set, achieving an MAE of 2.44 kcal mol^−1^. For molecules whose scaffolds differ from those represented in the dataset, predictive performance exhibited reduced accuracy (Fig. 3d and S17). These results indicate that the model performs robustly within its training scaffold domain, while its applicability to structurally distinct scaffolds remains more limited. A web-based platform has been established to enable users to predict ΔE* values of their ESIPT molecules using our optimized ML model (see SI, Section S2).

### Feature engineering revealing ESIPT key descriptors

Intramolecular hydrogen bond (H-bond) parameters, including bond lengths, angles, and energies, critically govern ESIPT reactivity. While empirical correlations between H-bond strengthening and ESIPT thermodynamics have been reported,^15,41,42,58–61^ the relative importance of individual or complex H-bond parameters contributing to these correlations remains unquantified. To address these gaps, we systematically evaluated the impact of H-bond parameters on ΔE* through data-driven interpretable ML across ∼700 O–H⋯O and O–H⋯N-type ESIPT systems.

**Fig. 4:**
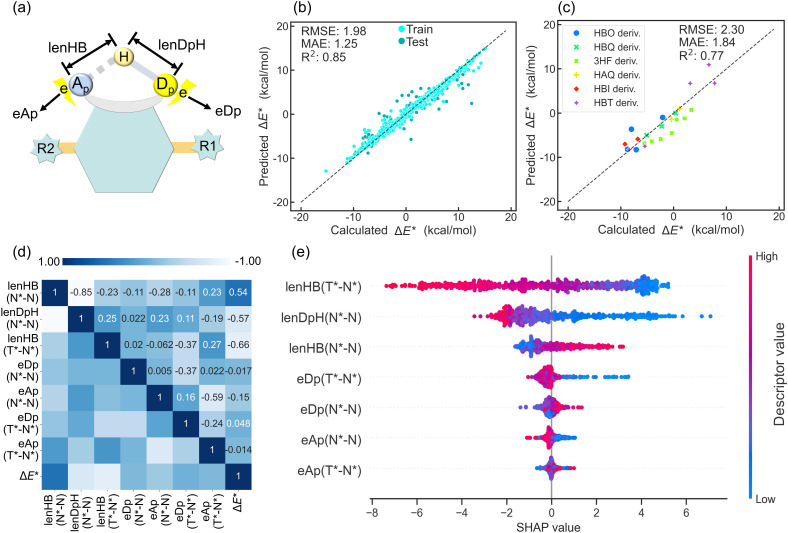
(a) Schematic representation of key parameters. (b) Predicted *versus* calculated ΔE* for the ET regressor model on the ESIPT dataset at one of the folds, and (c) validation on documented ESIPT molecules. (d) Pearson's correlation matrix among ΔE* and key descriptors. (e) SHAP summary plot of the ET regressor model, which visualizes the contribution of each descriptor to ΔE*. Each dot represents the SHAP value for a descriptor across the dataset, with the color indicating the actual value of the descriptors. Descriptors are ranked by the impact on ΔE*, with positive SHAP values driving higher ΔE* predictions and negative values indicating a reduction in ΔE*.

Previous studies have established that H-bond strength and the variation in electron populations on the proton donor and acceptor play pivotal roles in governing ESIPT behavior.^15,41^ Accordingly, feature engineering was used to extract key parameters for N and N* states: H-bond length (lenHB), proton donor-proton distance (lenDpH), and atomic dipole moment corrected Hirshfeld electron population^62^ for the proton donor (eDp) and acceptor (eAp) (Fig. 4a). For T* states, identical features except for lenDpH were obtained. To enhance the model's applicability to different H-bond types, preprocessing then generates three geometric differential descriptors: lenHB (N*–N), lenDpH (N*–N), and lenHB (T*–N*) and four electronic differential descriptors: eDp (N*–N), eAp (N*–N), eDp (T*–N*), and eAp (T*–N*), where “N*–N” or “T*–N*” denotes inter-state differential values. These key descriptors enable ΔE* prediction *via* the ML model. The extra trees (ET) model achieved optimal performance (5-fold CV MAE = 1.26 kcal mol^−1^) with one-fold MAE reaching 1.25 kcal mol^−1^. Test data points show tight alignment with the ideal prediction and exhibit a distribution similar to that of the training set (Fig. 4b). External validation on documented ESIPT molecules confirms the generalizability of above key descriptors (MAE = 1.82 kcal mol^−1^) (Fig. 4c).

Pearson correlation coefficient (*r*) analysis identified descriptors strongly correlated with ΔE* (Fig. 4d). Three geometric H-bond descriptors exhibited |*r*| > 0.5, indicating that H-bond geometric parameters play a primary role in determining ΔE* in ESIPT systems. Significantly, lenHB (N*–N) and lenDpH (N*–N) exhibit strong mutual anticorrelation (*r* = −0.85), while lenHB (T*–N*) shows minimal correlation with either descriptor, indicating its independence in predicting ΔE*. The four H-bond electronic descriptors exhibit weaker ΔE* correlation than geometric descriptors, indicating that ESICT plays a secondary role in modulating ΔE*. Notably, eAp (N*–N) shows the strongest correlation with ΔE* among electronic descriptors.

SHAP analysis was employed to interpret descriptor impacts on ΔE* (Fig. 4e). For individual descriptors, vertical point distribution manifests molecular count, while horizontal direction reflects the contribution of the descriptor value to the prediction result. Within the ESIPT dataset, lenHB (T*–N*) exhibits the strongest negative correlation with ΔE*, confirming that reduced H-bond energy leads to lower ΔE* and facilitates ESIPT. Furthermore, lenDpH (N*–N) and lenHB (N*–N) also significantly influence ΔE* with covalent bond length (lenDpH (N*–N)), demonstrating greater impact than H-bond length (lenHB (N*–N)). This suggests that covalent Dp–H bond modulation (*e.g.*, altering R groups from the EWG (*e.g.* tosyl group) to the EDG in N(R) H⋯N ESIPT systems)^15^ more effectively tunes ΔE* than H-bond adjustments. SHAP analysis further reveals opposing correlations: lenHB (N*–N) exhibits positive correlation with ΔE*, while lenDpH (N*–N) shows negative correlation. This indicates that excited-state H-bond strengthening favors exergonic ESIPT thermodynamics.^15^

In SHAP analysis, electronic descriptors exhibit narrower value distributions than H-bond descriptors, demonstrating that charge redistribution (ESICT) exerts less influence on ΔE* than H-bond parameters during ESIPT. We further observe a negative correlation between eAp (N*–N) and ΔE*, contrasting with the positive correlation for eDp (N*–N). This is consistent with our earlier findings,^41,58^ though initially validated in limited systems. Crucially, SHAP ranks eDp above eAp in descriptor importance, demonstrating that changes in proton donor charge dominate ΔE* determination. This proton donor-centric mechanism aligns with the greater influence of covalent Dp–H bond variations *versus* H-bond modifications during ESIPT. This may provide an explanation why S–H⋯O ESIPT systems^59,61^ sharing the 3HF derivative scaffold but differing in the proton donor exhibit fundamentally distinct ESIPT behavior from OH⋯O systems. Overall, the application of interpretable ML in the ESIPT dataset holds great significance for understanding the factors that influence ΔE*.

### Molecular generation

**Fig. 5:**
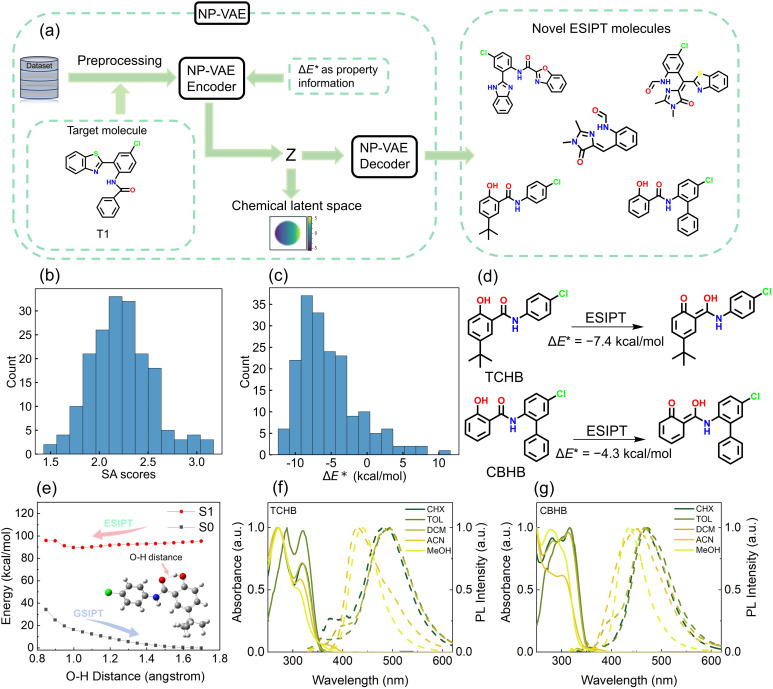
(a) The workflow of NPVAE for ESIPT molecule generation. Distribution of the (b) SA scores and (c) ΔE* for generated ESIPT molecules. (d) Schematic representation of the ESIPT process for TCHB and CBHB, where the prediction of ΔE* is marked. (e) Potential energy curves of the S_0_ and S_1_ states of TCHB along with the H-bond distance in a vacuum. The inset shows the stepwise scanned H-bond distance. Normalized steady-state absorption (solid line) and emission spectra (dash line) upon excitation at 310 nm of (f) TCHB and (g) CBHB in cyclohexane (CHX), toluene (TOL), dichloromethane (DCM), acetonitrile (ACN) and methanol (MeOH) at 298 K.

To explore novel ESIPT structures in an expanded chemical latent space, we implemented the NPVAE framework, a VAE developed by Ochiai *et al.* for molecular generation with an optimal combination of stability, reconstruction accuracy, and latent space organization. NPVAE's functional group-level preprocessing enables it to model large, structurally complex ESIPT molecules more accurately than atomic-level models, because it preserves essential structural motifs, including proton donor and acceptor groups. By retaining these functional groups during both training and generation, NPVAE exhibits a substantially higher probability of producing molecules with the characteristic features required for ESIPT, whereas atomic-level generative models often fail to capture or reproduce these critical functionalities.^36,51,52^ Trained on our constructed ESIPT dataset and documented ESIPT molecules, this framework was leveraged to design fluorescent probes targeting cell imaging applications. We selected a reported structurally minimal ESIPT probe^63^ (T1, Fig. 5a and S16) as our latent space navigation anchor, generating 182 novel analogs from its vicinity that potentially preserve T1-like properties (see SI Files, XLSX file 4 for generated ESIPT molecules). We also employed multiple anchor molecules to generate new structures, thereby demonstrating the model's capability to produce diverse molecular scaffolds (see SI, Section S3 and SI Files, XLSX file 5). The molecular generation workflow is depicted in Fig. 5a.

The SA scores and ADMET properties of the generated ESIPT molecules were predicted using ADMETlab 3.0.^64^ SA scores ranged from 1.5 to 3.5 (scale: 1 = easiest; scale 10 = hardest), indicating favorable synthetic feasibility through simple structural motifs and accessible routes (Fig. 5b). Subsequently, ΔE* values of generated ESIPT molecules were predicted using our XGBoost model combined with atom-pair descriptors (Fig. 5c). Given the increasing use of ESIPT fluorophores in bioimaging, intracellular sensing, and live-cell fluorescence studies,^1–5^ we further evaluated the generated ESIPT molecules using ADMET, log *S*, and log *D* to assess their potential suitability as fluorescent probes. (see SI Files, XLSX file 4).

Following multi-parametric evaluation (ΔE*, SA scores, ADMET, *etc.*), candidate ESIPT molecules were prioritized for experimental validation. Selection criteria emphasized: (i) thermodynamic favorability (ΔE* < 0), (ii) optimal safety/pharmacokinetic profiles, and (iii) high synthetic accessibility (SA) to ensure experimental feasibility. Balancing these factors, we identified two novel candidates: 5-(*tert*-butyl)-*N*-(4-chlorophenyl)-2-hydroxybenzamide (TCHB, SA = 1.67, ΔE* = −7.4 kcal mol^−1^) and *N*-(5-chloro-[1,1′-biphenyl]-2-yl)-2-hydroxybenzamide (CBHB, SA = 1.75, ΔE* = −4.3 kcal mol^−1^).

Their exergonic ΔE* values indicate ESIPT capability (Fig. 5d), and low SA scores suggest excellent synthetic accessibility. Both compounds are unreported in previous literature, confirming their novelty and potential for further exploration. Both compounds were synthesized for experimental validation (see SI, Section S4).

TD-DFT calculations performed at the theoretical level used to construct the ESIPT dataset yielded ΔE* values of −11.6 kcal mol^−1^ for TCHB and −10.4 kcal mol^−1^ for CBHB. Moreover, calculations using the M06-2X functional, which is known to better describe H-bond interactions, gave values of −12.9 kcal mol^−1^ and −12.3 kcal mol^−1^, respectively. These results also suggest that both molecules are capable of undergoing ESIPT. However, the predicted and calculated ΔE* values for TCHB and CBHB show a notable discrepancy, likely because their molecular scaffolds are not represented among the six types in the ESIPT dataset. The potential curves of TCHB and CBHB in S_0_ and S_1_ states were scanned based on constrained optimizations with varying O–H distances (Fig. 5e and S17). The results suggested a feature barrierless ESIPT (N* → T* isomerization) reactions. Steady-state spectroscopy (Table S2) in varied solvents showed N*/T* dual emission upon 310 nm excitation (Fig. 5f and g), directly evidencing photoinduced ESIPT. Crucially, the solvent-dependent N*/T* dual-emission ratio highlights the role of solvation in modulating ESIPT dynamics. These integrated theoretical and experimental results fully validate the AI-designed ESIPT molecules. Additionally, to validate the synthetic accessibility of other ESIPT candidates, concise synthetic routes with minimal steps were designed for several representative compounds (Schemes S1 and S2). Collectively, our models demonstrate high proficiency in identifying promising candidates in expansive chemical spaces, substantially outperforming traditional trial-and-error approaches in material development.

## Conclusions

In summary, we systematically constructed a high-quality ESIPT dataset by introducing ten substituents with progressively enhanced electron-donating abilities. Through a combination of qualitative descriptors and data-driven machine learning models, we achieved efficient and accurate prediction of ΔE*, significantly improving the throughput of high-efficiency ESIPT material screening. To enhance model interpretability, SHAP analysis was employed to quantify the contributions of key H-bond descriptors to ΔE* prediction. Furthermore, a variational autoencoder (VAE) was used to generate novel ESIPT molecules, which were subsequently filtered based on synthetic accessibility (SA) scores, predicted ΔE*, and ADMET properties. Notably, two AI-designed ESIPT molecules were successfully synthesized and experimentally validated, confirming the effectiveness of our data-driven molecular design strategy. Collectively, this work presents a robust and interpretable framework for ΔE* prediction and accelerates the discovery of novel, functional ESIPT materials.

## Author contributions

S. W., A. X., and Z. K. conceived the concepts. S. W., Z. K., and Z. Y. constructed the dataset, carried out the training and prediction of machine learning and machine learning models, and performed the interpretability analysis. C. Y., Y. L., and Y. G. designed and synthesized the TCHB and CBHB molecules, as well as the synthetic routes of other molecules. S. W. and H. Z. performed the quantum chemical calculations. Z. K. and A. X. polished the language and supervised this project. S. W. and Z. K. wrote this paper. All authors discussed the results and commented on the manuscript at all stages.

## Conflicts of interest

There are no conflicts to declare.

## Supplementary Material

SC-017-D5SC07051A-s001

SC-017-D5SC07051A-s002

SC-017-D5SC07051A-s003

SC-017-D5SC07051A-s004

SC-017-D5SC07051A-s005

SC-017-D5SC07051A-s006

## Data Availability

The data supporting this article have been included within the article or as part of the supplementary information (SI). Supplementary information: materials, synthesis details, calculation methods, and ML details (PDF); ESIPT dataset (XLSX file 1); documented ESIPT molecules (XLSX file 2); model selection through 5-fold CV and model configurations for various descriptors (XLSX file 3); generated ESIPT molecules and the corresponding properties (XLSX file 4); generated ESIPT molecules from multiple anchors (XLSX file 5). See DOI: https://doi.org/10.1039/d5sc07051a.

## References

[cit1] Demchenko A. P., Tang K.-C., Chou P.-T. (2013). Excited-state proton coupled charge transfer modulated by molecular structure and media polarization. Chem. Soc. Rev..

[cit2] Tang L., Shi J., Huang Z., Yan X., Zhang Q., Zhong K., Hou S., Bian Y. (2016). An ESIPT-based fluorescent probe for selective detection of homocysteine and its application in live-cell imaging. Tetrahedron Lett..

[cit3] He L., Dong B., Liu Y., Lin W. (2016). Fluorescent chemosensors manipulated by dual/triple interplaying sensing mechanisms. Chem. Soc. Rev..

[cit4] Shynkar V. V., Klymchenko A. S., Kunzelmann C., Duportail G., Muller C. D., Demchenko A. P., Freyssinet J.-M., Mely Y. (2007). Fluorescent Biomembrane Probe for Ratiometric Detection of Apoptosis. J. Am. Chem. Soc..

[cit5] Oncul S., Klymchenko A. S., Kucherak O. A., Demchenko A. P., Martin S., Dontenwill M., Arntz Y., Didier P., Duportail G., Mély Y. (2010). Liquid ordered phase in cell membranes evidenced by a hydration-sensitive probe: Effects of cholesterol depletion and apoptosis. Biochim. Biophys. Acta.

[cit6] Tang K.-C., Chang M.-J., Lin T.-Y., Pan H.-A., Fang T.-C., Chen K.-Y., Hung W.-Y., Hsu Y.-H., Chou P.-T. (2011). Fine Tuning the Energetics of Excited-State Intramolecular Proton Transfer (ESIPT): White Light Generation in A Single ESIPT System. J. Am. Chem. Soc..

[cit7] Azarias C., Budzák Š., Laurent A. D., Ulrich G., Jacquemin D. (2016). Tuning ESIPT fluorophores into dual emitters. Chem. Sci..

[cit8] Padalkar V. S., Seki S. (2016). Excited-state intramolecular proton-transfer (ESIPT)-inspired solid state emitters. Chem. Soc. Rev..

[cit9] Zhang Z., Chen Y.-A., Hung W.-Y., Tang W.-F., Hsu Y.-H., Chen C.-L., Meng F.-Y., Chou P.-T. (2016). Control of the Reversibility of Excited-State Intramolecular Proton Transfer (ESIPT) Reaction: Host-Polarity Tuning White Organic Light Emitting Diode on a New Thiazolo[5,4-d]thiazole ESIPT System. Chem. Mater..

[cit10] Benelhadj K., Muzuzu W., Massue J., Retailleau P., Charaf-Eddin A., Laurent A. D., Jacquemin D., Ulrich G., Ziessel R. (2014). White Emitters by Tuning the Excited-State Intramolecular Proton-Transfer Fluorescence Emission in 2-(2′-Hydroxybenzofuran)benzoxazole Dyes. Chem.–Eur. J..

[cit11] Kwon J. E., Park S. Y. (2011). Advanced Organic Optoelectronic Materials: Harnessing Excited-State Intramolecular Proton Transfer (ESIPT) Process. Adv. Mater..

[cit12] Wu X., Wang C.-H., Ni S., Wu C.-C., Lin Y.-D., Qu H.-T., Wu Z.-H., Liu D., Yang M.-Z., Su S.-J., Zhu W., Chen K., Jiang Z.-C., Yang S.-D., Hung W.-Y., Chou P.-T. (2024). Multiple Enol–Keto Isomerization and Excited-State Unidirectional Intramolecular Proton Transfer Generate Intense, Narrowband Red OLEDs. J. Am. Chem. Soc..

[cit13] Che Z.-L., Yu Y.-J., Yan C.-C., Ge S.-J., Zuo P., Wu J.-J., Liu F.-M., Feng Z.-Q., Liao L.-S., Wang X.-D. (2025). Advancing Beyond 800 Nm: Highly Stable Near-Infrared Thermally Activated Delayed Lasing Triggered by Excited-State Intramolecular Proton Transfer Process. Adv. Mater..

[cit14] Tao M., Wen L., Huo D., Kuang Z., Song D., Wan Y., Zhao H., Yan J., Xia A. (2021). Solvent Effect on Excited-State Intramolecular Proton-Coupled Charge Transfer Reaction in Two Seven-Membered Ring Pyrrole-Indole Hydrogen Bond Systems. J. Phys. Chem. B.

[cit15] Tseng H. W., Liu J. Q., Chen Y. A., Chao C. M., Liu K. M., Chen C. L., Lin T. C., Hung C. H., Chou Y. L., Lin T. C., Wang T. L., Chou P. T. (2015). Harnessing Excited-State Intramolecular Proton-Transfer Reaction via a Series of Amino-Type Hydrogen-Bonding Molecules. J. Phys. Chem. Lett..

[cit16] Kuang Z., Guo Q., Wang X., Song H., Maroncelli M., Xia A. (2018). Ultrafast Ground-State Intramolecular Proton Transfer in Diethylaminohydroxyflavone Resolved with Pump-Dump-Probe Spectroscopy. J. Phys. Chem. Lett..

[cit17] Liu Z. Y., Wei Y. C., Chou P. T. (2021). Correlation between Kinetics and Thermodynamics for Excited-State Intramolecular Proton Transfer Reactions. J. Phys. Chem. A.

[cit18] Shi Y., Shi H., Zhang Y., Zang X., Zhao Z., Zhao S., Qiao B., Liang Z., Xu Z., Wang L., Song D. (2024). Identifying the Quantitative Relationship Between the Molecular Structure and the Horizontal Transition Dipole Orientation of TADF Emitters. Adv. Opt. Mater..

[cit19] Kim H. S., Cheon H. J., Lee S. H., Kim J., Yoo S., Kim Y.-H., Adachi C. (2025). Advancing efficiency in deep-blue OLEDs: Exploring a machine learning–driven multiresonance TADF molecular design. Sci. Adv..

[cit20] Gao Y.-C., Yao N., Chen X., Yu L., Zhang R., Zhang Q. (2023). Data-Driven Insight into the Reductive Stability of Ion–Solvent Complexes in Lithium Battery Electrolytes. J. Am. Chem. Soc..

[cit21] Gao Y.-C., Yuan Y.-H., Huang S., Yao N., Yu L., Chen Y.-P., Zhang Q., Chen X. (2025). A Knowledge–Data Dual-Driven Framework for Predicting the Molecular Properties of Rechargeable Battery Electrolytes. Angew. Chem., Int. Ed..

[cit22] Xu S., Liu X., Cai P., Li J., Wang X., Liu B. (2022). Machine-Learning-Assisted Accurate Prediction of Molecular Optical Properties upon Aggregation. Adv. Sci..

[cit23] Zhu L., Huang M., Han G., Wei Z., Yi Y. (2025). The Key Descriptors for Predicting the Exciton Binding Energy of Organic Photovoltaic Materials. Angew. Chem., Int. Ed..

[cit24] Zhang S., Li S., Song S., Zhao Y., Gao L., Chen H., Li H., Lin J. (2025). Deep Learning-Assisted Design of Novel Donor–Acceptor Combinations for Organic Photovoltaic Materials with Enhanced Efficiency. Adv. Mater..

[cit25] Zhang Z.-Y., Liu X., Shen L., Chen L., Fang W.-H. (2021). Machine Learning with Multilevel Descriptors for Screening of Inorganic Nonlinear Optical Crystals. J. Phys. Chem. C.

[cit26] Yu Z., Xue P., Xie B.-B., Shen L., Fang W.-H. (2024). Multi-fidelity machine learning for predicting bandgaps of nonlinear optical crystals. Phys. Chem. Chem. Phys..

[cit27] Huang W., Huang S., Fang Y., Zhu T., Chu F., Liu Q., Yu K., Chen F., Dong J., Zeng W. (2024). AI-Powered Mining of Highly Customized and Superior ESIPT-Based Fluorescent Probes. Adv. Sci..

[cit28] Heid E., Greenman K. P., Chung Y., Li S.-C., Graff D. E., Vermeire F. H., Wu H., Green W. H., McGill C. J. (2024). Chemprop: A Machine Learning Package for Chemical Property Prediction. J. Chem. Inf. Model..

[cit29] Pan X., Wang H., Li C., Zhang J. Z. H., Ji C. (2021). MolGpka: A Web Server for Small Molecule pKa Prediction Using a Graph-Convolutional Neural Network. J. Chem. Inf. Model..

[cit30] Ren F., Aliper A., Chen J., Zhao H., Rao S., Kuppe C., Ozerov I. V., Zhang M., Witte K., Kruse C., Aladinskiy V., Ivanenkov Y., Polykovskiy D., Fu Y., Babin E., Qiao J., Liang X., Mou Z., Wang H., Pun F. W., Torres-Ayuso P., Veviorskiy A., Song D., Liu S., Zhang B., Naumov V., Ding X., Kukharenko A., Izumchenko E., Zhavoronkov A. (2025). A small-molecule TNIK inhibitor targets fibrosis in preclinical and clinical models. Nat. Biotechnol..

[cit31] Stokes J. M., Yang K., Swanson K., Jin W., Cubillos-Ruiz A., Donghia N. M., MacNair C. R., French S., Carfrae L. A., Bloom-Ackermann Z., Tran V. M., Chiappino-Pepe A., Badran A. H., Andrews I. W., Chory E. J., Church G. M., Brown E. D., Jaakkola T. S., Barzilay R., Collins J. J. (2020). A Deep Learning Approach to Antibiotic Discovery. Cell.

[cit32] Liu G., Catacutan D. B., Rathod K., Swanson K., Jin W., Mohammed J. C., Chiappino-Pepe A., Syed S. A., Fragis M., Rachwalski K., Magolan J., Surette M. G., Coombes B. K., Jaakkola T., Barzilay R., Collins J. J., Stokes J. M. (2023). Deep learning-guided discovery of an antibiotic targeting Acinetobacter baumannii. Nat. Chem. Biol..

[cit33] Moret M., Friedrich L., Grisoni F., Merk D., Schneider G. (2020). Generative molecular design in low data regimes. Nat. Mach. Intell..

[cit34] Walters W. P., Barzilay R. (2021). Applications of Deep Learning in Molecule Generation and Molecular Property Prediction. Acc. Chem. Res..

[cit35] Swanson K., Liu G., Catacutan D. B., Arnold A., Zou J., Stokes J. M. (2024). Generative AI for designing and validating easily synthesizable and structurally novel antibiotics. Nat. Mach. Intell..

[cit36] Ochiai T., Inukai T., Akiyama M., Furui K., Ohue M., Matsumori N., Inuki S., Uesugi M., Sunazuka T., Kikuchi K., Kakeya H., Sakakibara Y. (2023). Variational autoencoder-based chemical latent space for large molecular structures with 3D complexity. Commun. Chem..

[cit37] Niu X., Dang Y., Sun Y., Hu W. (2023). Judicious training pattern for superior molecular reorganization energy prediction model. J. Energy Chem..

[cit38] Dollar O., Joshi N., Pfaendtner J., Beck D. A. C. (2023). Efficient 3D Molecular Design with an E(3) Invariant Transformer VAE. J. Phys. Chem. A.

[cit39] Runcie N. T., Mey A. S. J. S. (2023). SILVR: Guided Diffusion for Molecule Generation. J. Chem. Inf. Model..

[cit40] Raucci U. (2025). Capturing Excited State Proton Transfer Dynamics with Reactive Machine Learning Potentials. J. Phys. Chem. Lett..

[cit41] Tao M., Li Y., Huang Q., Zhao H., Lan J., Wan Y., Kuang Z., Xia A. (2022). Correlation between Excited-State Intramolecular Proton Transfer and Electron Population on Proton Donor/Acceptor in 2-(2′-Hydroxyphenyl)oxazole Derivatives. J. Phys. Chem. Lett..

[cit42] Liu Z.-Y., Hu J.-W., Huang T.-H., Chen K.-Y., Chou P.-T. (2020). Excited-state intramolecular proton transfer in the kinetic-control regime. Phys. Chem. Chem. Phys..

[cit43] Liu Z.-Y., Hu J.-W., Chen C.-L., Chen Y.-A., Chen K.-Y., Chou P.-T. (2018). Correlation among Hydrogen Bond, Excited-State Intramolecular Proton-Transfer Kinetics and Thermodynamics for –OH Type Proton-Donor Molecules. J. Phys. Chem. C.

[cit44] Chen C.-L., Tseng H.-W., Chen Y.-A., Liu J.-Q., Chao C.-M., Liu K.-M., Lin T.-C., Hung C.-H., Chou Y.-L., Lin T.-C., Chou P.-T. (2016). Insight into the Amino-Type Excited-State Intramolecular Proton Transfer Cycle Using N-Tosyl Derivatives of 2-(2′-Aminophenyl)benzothiazole. J. Phys. Chem. A.

[cit45] Chung M.-W., Liao J.-L., Tang K.-C., Hsieh C.-C., Lin T.-Y., Liu C., Lee G.-H., Chi Y., Chou P.-T. (2012). Structural tuning intra- versus inter-molecular proton transfer reaction in the excited state. Phys. Chem. Chem. Phys..

[cit46] Lin T.-Y., Tang K.-C., Yang S.-H., Shen J.-Y., Cheng Y.-M., Pan H.-A., Chi Y., Chou P.-T. (2012). The Empirical Correlation between Hydrogen Bonding Strength and Excited-State Intramolecular Proton Transfer in 2-Pyridyl Pyrazoles. J. Phys. Chem. A.

[cit47] Landrum G. , Rdkit: Open-Source Cheminformatics, 2010

[cit48] Ramsundar B. , Eastman P., Walters P. and Pande V., Deep Learning for the Life Sciences: Applying Deep Learning to Genomics, Microscopy, Drug Discovery, and More, Deep Learning for the Life Sciences: Applying Deep Learning to Genomics, Microscopy, Drug Discovery, and More, 2019

[cit49] Van der Maaten L., Hinton G. (2008). Visualizing data using t-SNE. J. Mach. Learn. Res..

[cit50] Chen H., Covert I. C., Lundberg S. M., Lee S.-I. (2023). Algorithms to estimate Shapley value feature attributions. Nat. Mach. Intell..

[cit51] Jin W., Barzilay R., Jaakkola T. (2018). Junction Tree Variational Autoencoder for Molecular Graph Generation. ICML.

[cit52] Jin W. , Barzilay D. R. and Jaakkola T., Hierarchical Generation of Molecular Graphs using Structural Motifs, Proceedings of the 37th International Conference on Machine Learning, 2020, vol. 119, pp. 4839–4848

[cit53] Rogers D., Hahn M. (2010). Extended-Connectivity Fingerprints. J. Chem. Inf. Model..

[cit54] Smith T. P., Zaklika K. A., Thakur K., Walker G. C., Tominaga K., Barbara P. F. (1991). Spectroscopic studies of excited-state intramolecular proton transfer in 1-(acylamino)anthraquinones. J. Phys. Chem..

[cit55] Choi J. R., Jeoung S. C., Cho D. W. (2004). Two-photon-induced excited-state intramolecular proton transfer process in 1-hydroxyanthraquinone. Chem. Phys. Lett..

[cit56] Nagaoka S.-i., Nagashima U. (1996). Effects of node of wave function upon excited-state intramolecular proton transfer of hydroxyanthraquinones and aminoanthraquinones. Chem. Phys..

[cit57] Carhart R. E., Smith D. H., Venkataraghavan R. (1985). Atom pairs as molecular features in structure-activity studies: definition and applications. J. Chem. Inf. Comput. Sci..

[cit58] Wei S., Situ Z., Zhang J., Li Y., Wan Y., Zhao H., Lan J., Kuang Z., Xia A. (2025). Ultrafast Proton-Coupled Electron-Transfer Dynamics in Amino-Type Indole-Triazolopyrimidine Derivatives. J. Phys. Chem. Lett..

[cit59] Wang C.-H., Liu Z.-Y., Huang C.-H., Chen C.-T., Meng F.-Y., Liao Y.-C., Liu Y.-H., Chang C.-C., Li E. Y., Chou P.-T. (2021). Chapter Open for the Excited-State Intramolecular Thiol Proton Transfer in the Room-Temperature Solution. J. Am. Chem. Soc..

[cit60] Chen C. L., Chen Y. T., Demchenko A. P., Chou P. T. (2018). Amino proton donors in excited-state intramolecular proton-transfer reactions. Nat. Rev. Chem..

[cit61] Wang J.-K., Wang C.-H., Wu C.-C., Chang K.-H., Wang C.-H., Liu Y.-H., Chen C.-T., Chou P.-T. (2024). Hydrogen-Bonded Thiol Undergoes Unconventional Excited-State Intramolecular Proton-Transfer Reactions. J. Am. Chem. Soc..

[cit62] Lu T., Chen F. (2012). Atomic dipole moment corrected hirshfeld population method. J. Theor. Comput. Chem..

[cit63] Huang S., Feng B., Cheng X., Huang X., Ding J., Yu K., Dong J., Zeng W. (2023). Controlling ESIPT-based AIE effects for designing optical materials with single-component white-light emission. Chem. Eng. J..

[cit64] Fu L., Shi S., Yi J., Wang N., He Y., Wu Z., Peng J., Deng Y., Wang W., Wu C., Lyu A., Zeng X., Zhao W., Hou T., Cao D. (2024). ADMETlab 3.0: an updated comprehensive online ADMET prediction platform enhanced with broader coverage, improved performance, API functionality and decision support. Nucleic Acids Res..

