# Supplementary material for: Unveiling key descriptors via machine learning: toward rational molecular design of chromophores with excited-state intramolecular proton transfer
Source: Chem Sci. 2026 Feb 9;17(14):7146–56. doi: 10.1039/d5sc07051a (PMC12910287; doi:10.1039/d5sc07051a)
Supplement: SC-017-D5SC07051A-s006 [file SC-017-D5SC07051A-s006.pdf]

Supplementary Information (SI) for Chemical Science.
This journal is © The Royal Society of Chemistry 2026

Supporting information

Unveiling Key Descriptors via Machine Learning: Toward Rational Molecular Design of Chromophores with Excited-State Intramolecular Proton Transfer

Shengsheng Wei,[1] Zipeng Yang,[1] Chao Yang,[1] Hongmei Zhao,[1] Yang Li,[1] Yuanyuan Guo,[1] Andong Xia,[1] Zhuoran Kuang,[1*]*

1. State Key Laboratory of Information Photonic and Optical Communications, and School of Science, Beijing University of Posts and Telecommunications (BUPT), Beijing 100876, P. R. China

AUTHOR INFORMATION

Corresponding Authors

*Andong Xia E-mail: andongxia@bupt.edu.cn

*Zhuoran Kuang E-mail: kuang@bupt.edu.cn

Table of Contents

S1. Methods....3

S1.1 Construction of ESIPT Dataset....3

S1.2 Prediction Models....7

1. ML Algorithms....7

2. Graph-Based Models....19

S1.3 Molecular Fingerprint....19

1. RDKit Fingerprints....19

2. Atom-Pair and Topological Torsion Fingerprints[4]. 19

3. ECFP....20

4. Quantitative Descriptors....21

5. MACCS....29

6. DMPNN....39

S1.4 Clustering Analysis and Visualization....40

S1.5 ADMET Evaluation....44

S2. ΔE^* Prediction Website....45

S3. Molecular Generation from NPVAE....46

S4. Materials and Instruments....51

S5. References....58

S1. Methods

S1.1 Construction of ESIPT Dataset

The geometries of the singlet ground states were optimized using density functional theory (DFT) with the B3LYP hybrid functional in vacuum. The electronic transition properties of all molecules were computed via time-dependent density functional theory (TD-DFT) at the same level of theory. The 6-31G(d,p) basis set was applied to all atoms. Additionally, in constructing the ESIPT dataset, the lowest singlet excited-state (S1) geometries of both the N* and T* states were optimized using TD-B3LYP with the 6-31G(d,p) basis set in vacuum. However, the N* and T* geometries of some molecules failed to converge. Therefore, although the theoretical number of molecules is 6 × 11 × 11 = 726, we ultimately obtained only 704 converged structures. ΔE^* calculation for TCHB and CBHB were computed in vacuum at the M06-2X and B3LYPry. Potential energy curves for the S_0 and S_1 states of TCHB were also computed in vacuum using the relaxed scan method at the M06-2X/6-31G(d,p) level of theory, with the H-bonding distance as the reaction coordinate. All DFT and TD-DFT calculations were carried out using the Gaussian 16 software package[1] on the CPU cluster at the Supercomputing Center of Beijing University of Posts and Telecommunications.

Atomic Dipole-Corrected Hirshfeld (ADCH) charges are a modified version of the traditional Hirshfeld atomic charges, developed to overcome the well-known underestimation of charge separation in polar systems. In the conventional Hirshfeld method, the electron density of a molecule is partitioned among its constituent atoms based on their respective promolecular densities, which often leads to unrealistically low atomic charges due to its reliance on the neutral atom reference.

To address this limitation, the ADCH method introduces a correction based on atomic dipole moments. Specifically, it includes an additional term that accounts for the local dipole contribution of each atom, thereby improving the physical accuracy of the charge distribution. The resulting charges are more responsive to changes in the electronic environment and correlate better with electrostatic potentials and experimental data. ADCH charges retain the computational efficiency and simplicity of the original Hirshfeld approach, while providing a

more realistic description of electron distribution, especially in systems with significant intramolecular polarization or charge transfer.

Regarding the selection of parent molecules, we first gathered 18 ESIPT parent molecules from the literature. However, not all of these molecules undergo ESIPT. For instance, parent molecules with weak hydrogen bonds, such as NH···N, have a low probability of exhibiting ESIPT. To prevent the issue of generating derivatives that do not undergo ESIPT, we excluded those with weak NH···N hydrogen bonds. Instead, we focused on parent molecules with stronger hydrogen bonds, such as OH···N or OH···O, ensuring that the selected molecules inherently possess ultrafast ESIPT properties. Furthermore, we subsequently selected those parent molecules that have been reported to possess promising application potential. As a result, we narrowed our selection to six well-documented parent structures (HBO, HBQ, HBI, HBT, 3HF, and HAQ), thereby ensuring the stability and reliability of their ESIPT behavior.

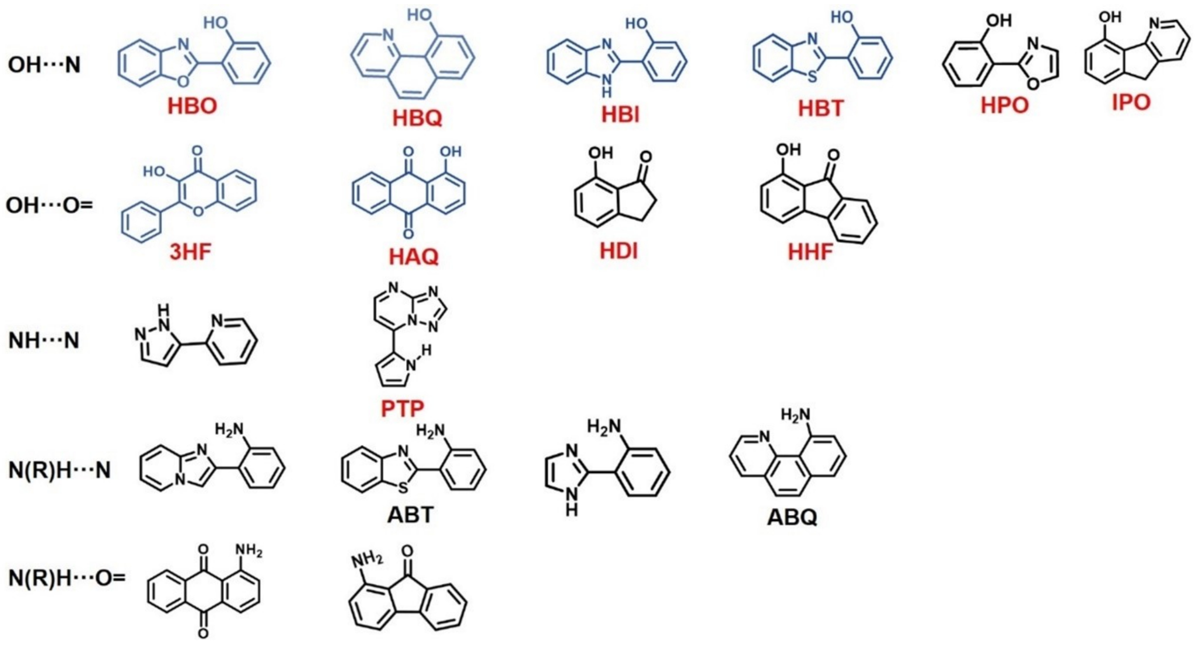

Figure S1. ESIPT parent molecules collected from literatures. The molecules colored in blue are used to construct the ESIPT dataset. The text directly below the molecules is their commonly used abbreviation in the literature.

Table S1. Calculated energy of N* and T* state for 11 ESIPT parent molecules.

	Energy of N* (kal/mol)	Energy of T* (kcal/mol)	ΔE* (kcal/mol)
3HF	-504172.2276	-504181.81	-9.58255774
HAQ	-479525.1906	-479522.78	2.41490874
HBI	-430637.9363	-430647.1	-9.164535436
HBO	-443100.0625	-443105.61	-5.543364238
HBT	-645780.8001	-645786.27	-5.468524007
HBQ	Only Converges to T*		
HHF	-408377.845	-408372.63	5.210470507
HPO	-346644.6426	-346649.34	-4.695638294
PTP	-389674.415	-389664.51	9.900428352
IPO	-371961.5322	-371983.69	-22.15442755
HDI	-312674.7829	-312675.68	-0.901618291

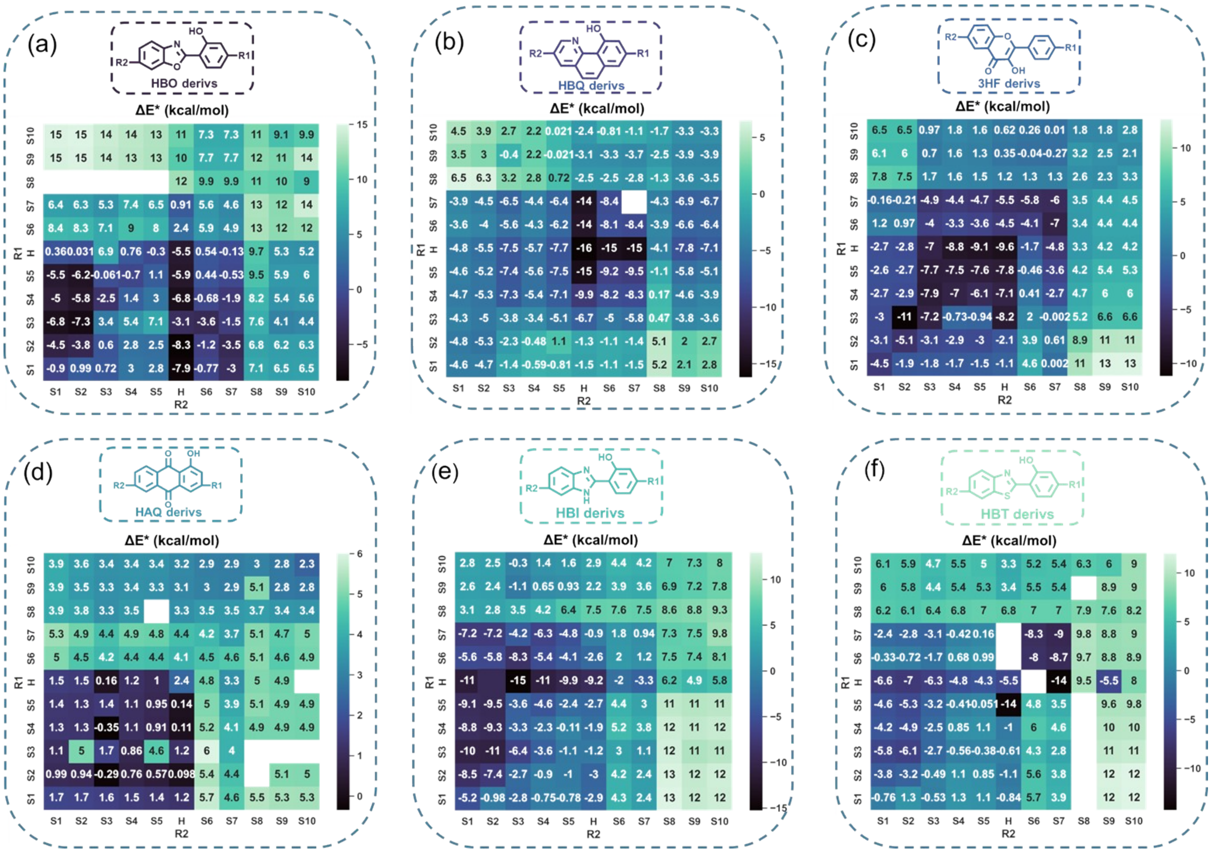

Figure S2. Heatmap plots of ΔE^* for derivatives of (a) HBO, (b) HBQ, (c) 3HF, (d) HAQ, (e) HBI, and (f) HBT. The white grid indicates that attempts to optimize the molecular geometries in their N* (T*) state typically result in the corresponding T* (N*) structure instead.

S1.2 Prediction Models

The hold-out method is used to evaluate the optimal model. Training data were split into five parts, four parts as sub-training, and one portion as validation. Five fold cross-validation (CV) is used for model selection. Details of these model configurations and five fold CV can be found in supporting information xlsx file 2. The experiment of machine learning and graph model was performed on CPU.

1. ML Algorithms

Ten machine learning algorithms were utilized to predict the ΔE^* of ESIPT molecules. All machine learning algorithm implementations come from the scikit-learn package,[2] including extreme gradient boosting (XGBoost), extra trees (ET), bagging, random forests (RF), adaptive boosting (AdaBoost), gradient boosting (GB), k-nearest neighbors (KNN), decision tree (DT), and supporting vector machine (SVM). Multilayer Perceptron (MLP), a type of neural network architecture, was implemented using the TensorFlow framework.[3]

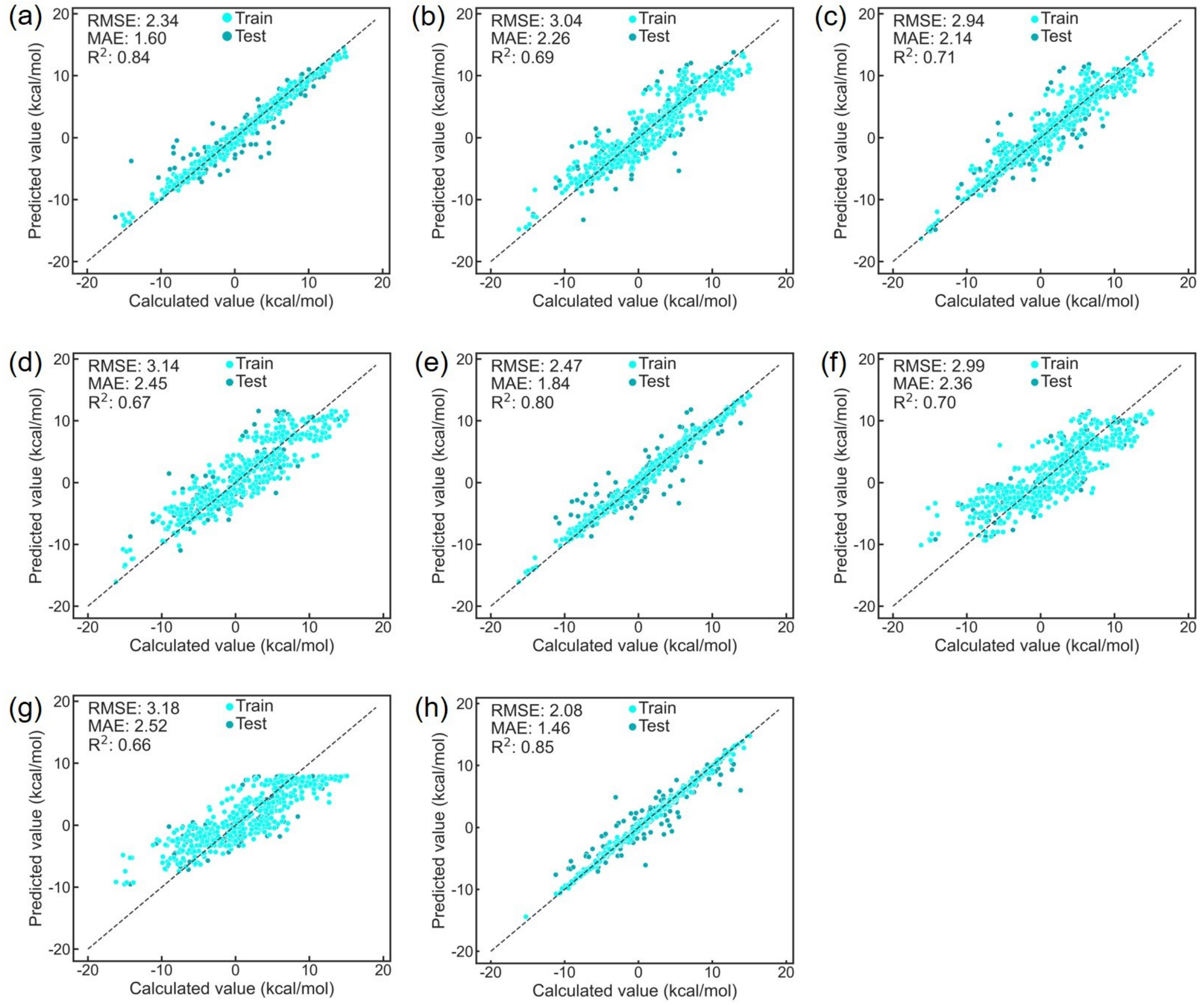

Figure S3. The plot of predicted versus calculated ΔE^* of (a) AtomPair, (b) ECFP, (c) RDKit, (d) Topological torsion, (e) Quantitative descriptor, (f) MACCS, (g) DMPNN, (h) Feature engineering descriptors used XGBoost model in CV at one of folds.

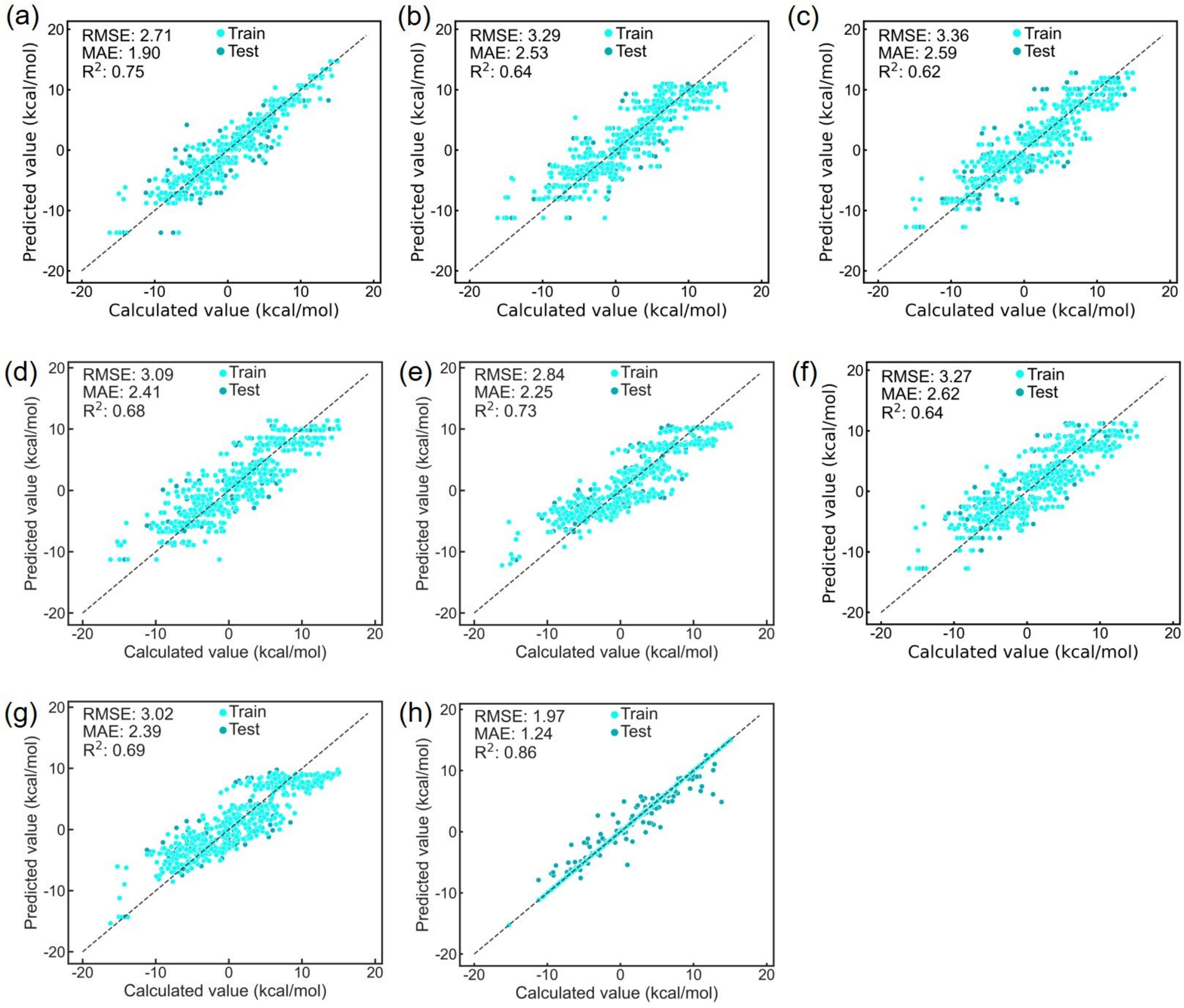

Figure S4. The plot of predicted versus calculated ΔE^* of (a) AtomPair, (b) ECFP, (c) RDKit, (d) Topological torsion, (e) Quantitative descriptor, (f) MACCS, (g) DMPNN, (h) Feature engineering descriptors used ET model in CV at one of folds.

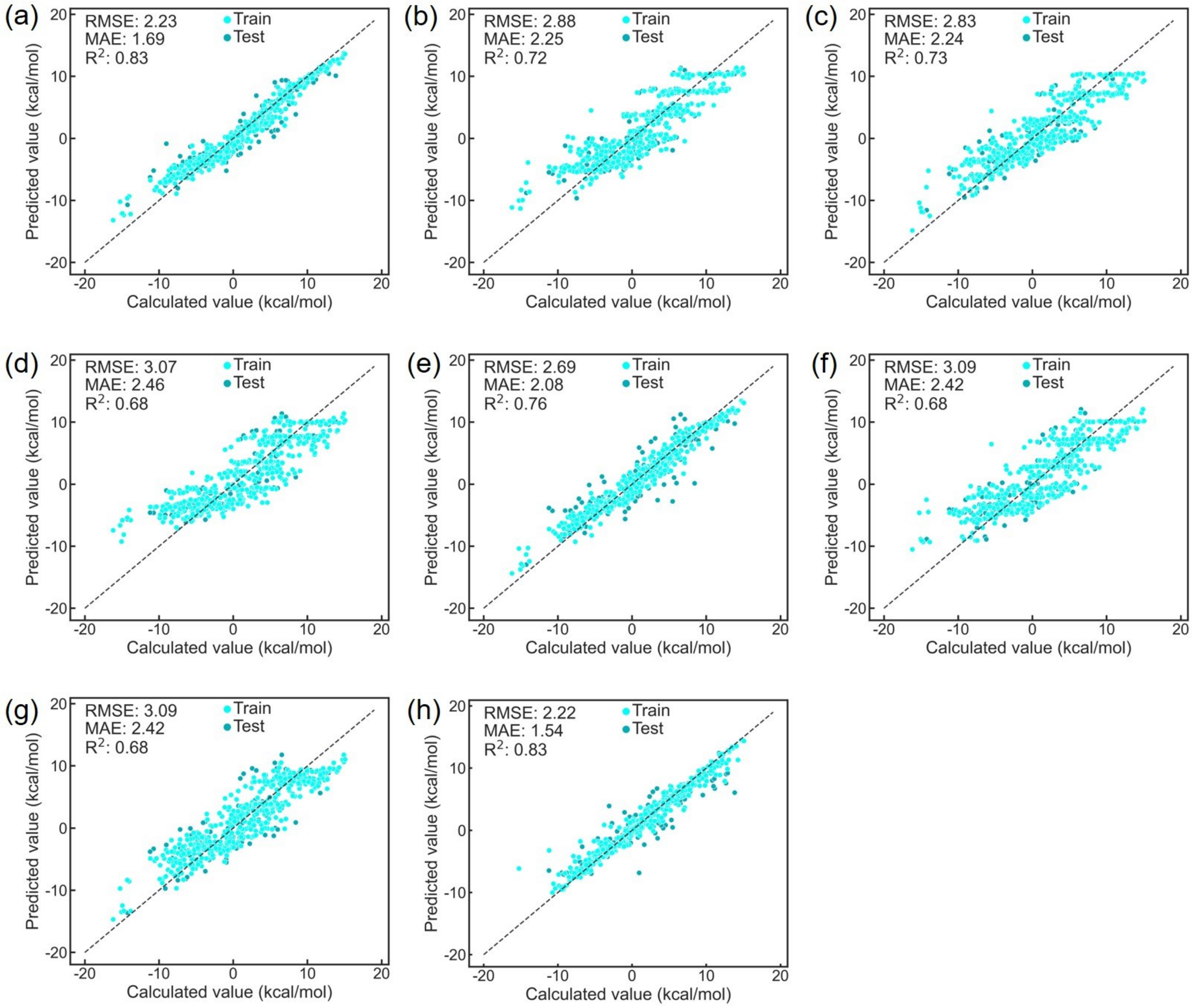

Figure S5. The plot of predicted versus calculated ΔE^* of (a) Atom-pair, (b) ECFP, (c) RDKit, (d) Topological torsion, (e) Quantitative descriptor, (f) MACCS, (g) DMPNN, (h) Feature engineering descriptors used Bagging model in CV at one of folds.

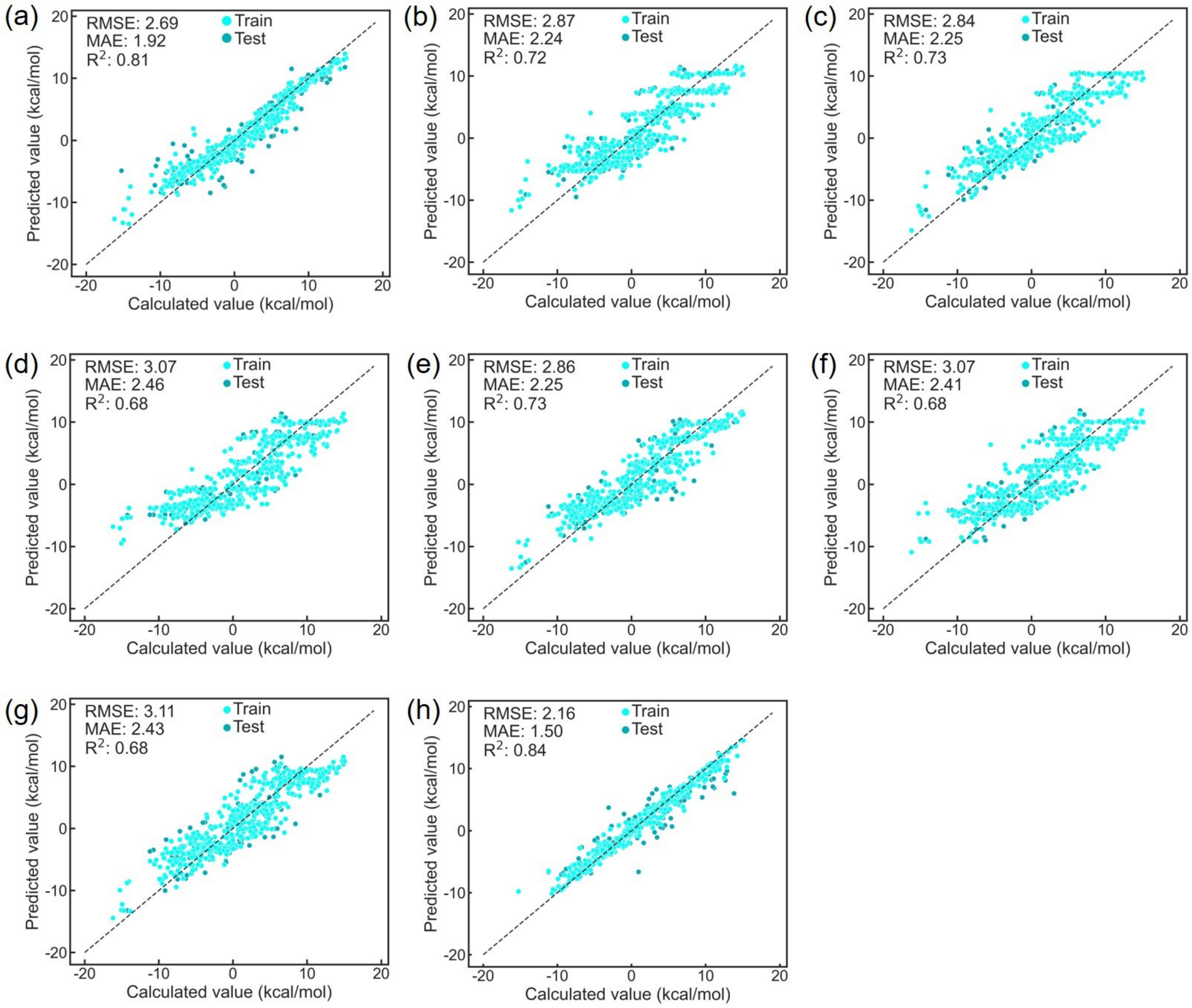

Figure S6. The plot of predicted versus calculated ΔE^* of (a) AtomPair, (b) ECFP, (c) RDKit, (d) Topological torsion, (e) Quantitative descriptor, (f) MACCS, (g) DMPNN, (h) Feature engineering descriptors used RF model in CV at one of folds.

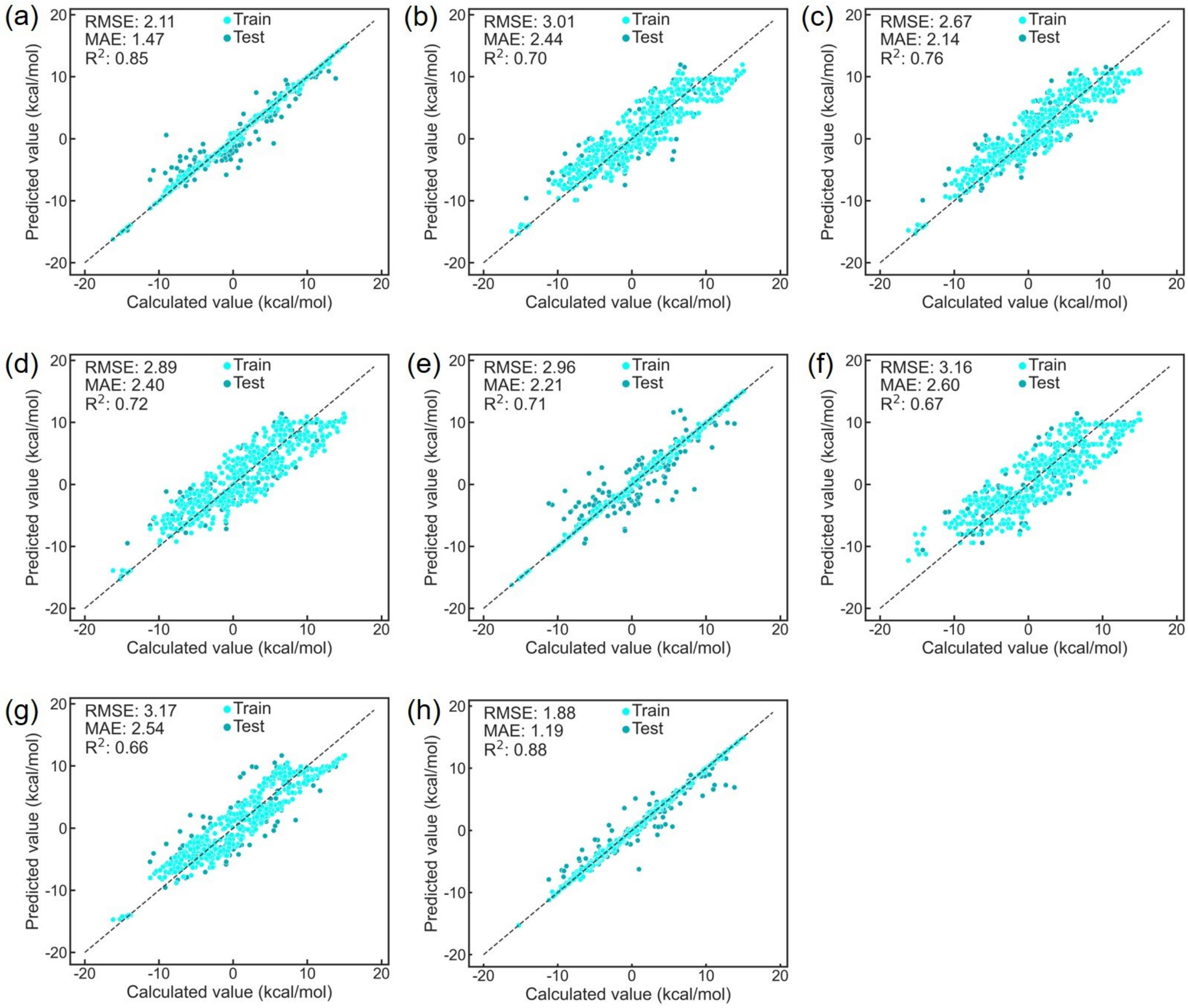

Figure S7. The plot of predicted versus calculated $\Delta E*$ of (a) AtomPair, (b) ECFP, (c) RDKit, (d) Topological torsion, (e) Quantitative descriptor, (f) MACCS, (g) DMPNN, (h) Feature engineering descriptors used AdaBoost model in CV at one of folds.

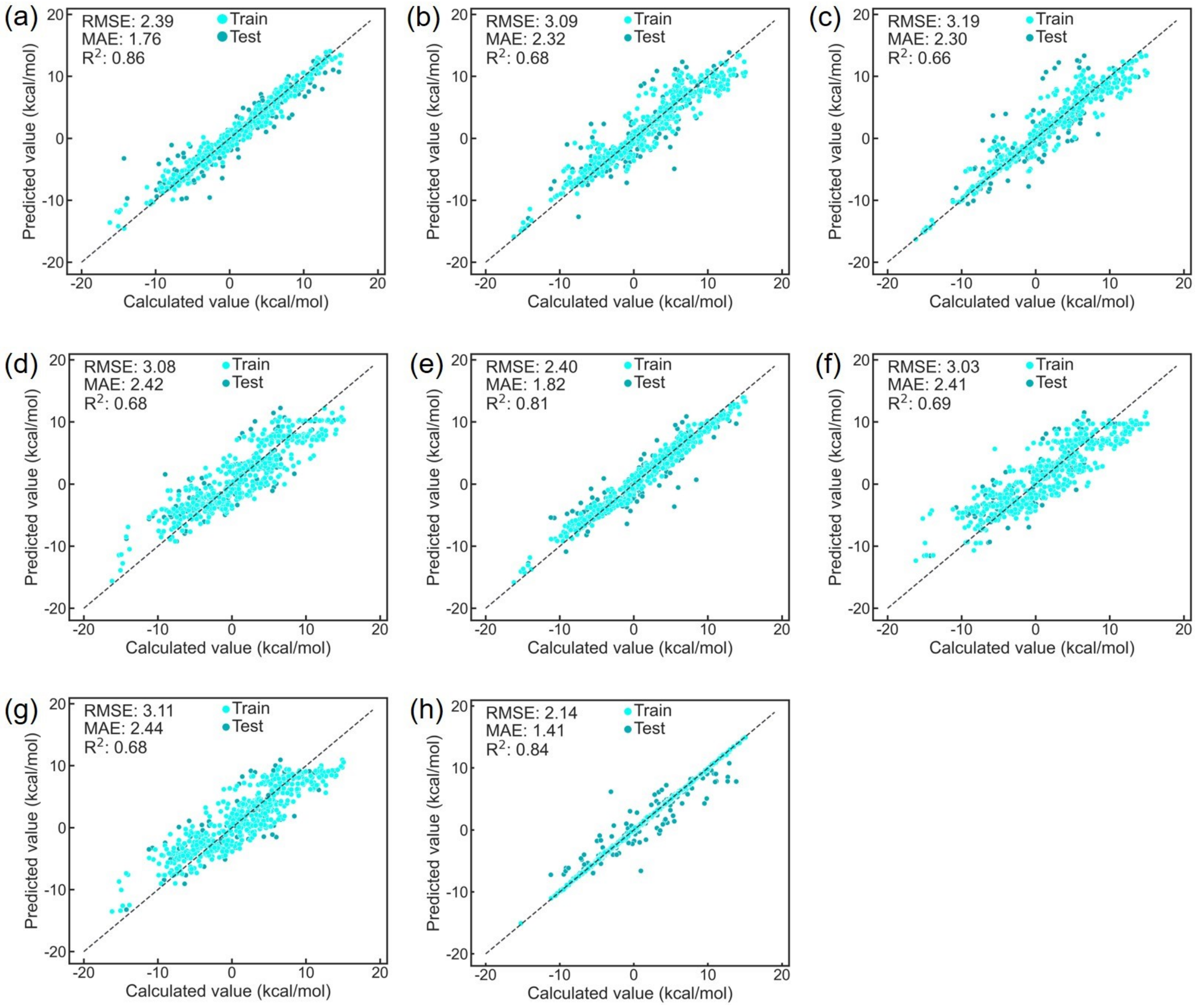

Figure S8. The plot of predicted versus calculated ΔE^* of (a) AtomPair, (b) ECFP, (c) RDKit, (d) Topological torsion, (e) Quantitative descriptor, (f) MACCS, (g) DMPNN, (h) Feature engineering descriptors used GB model in CV at one of folds.

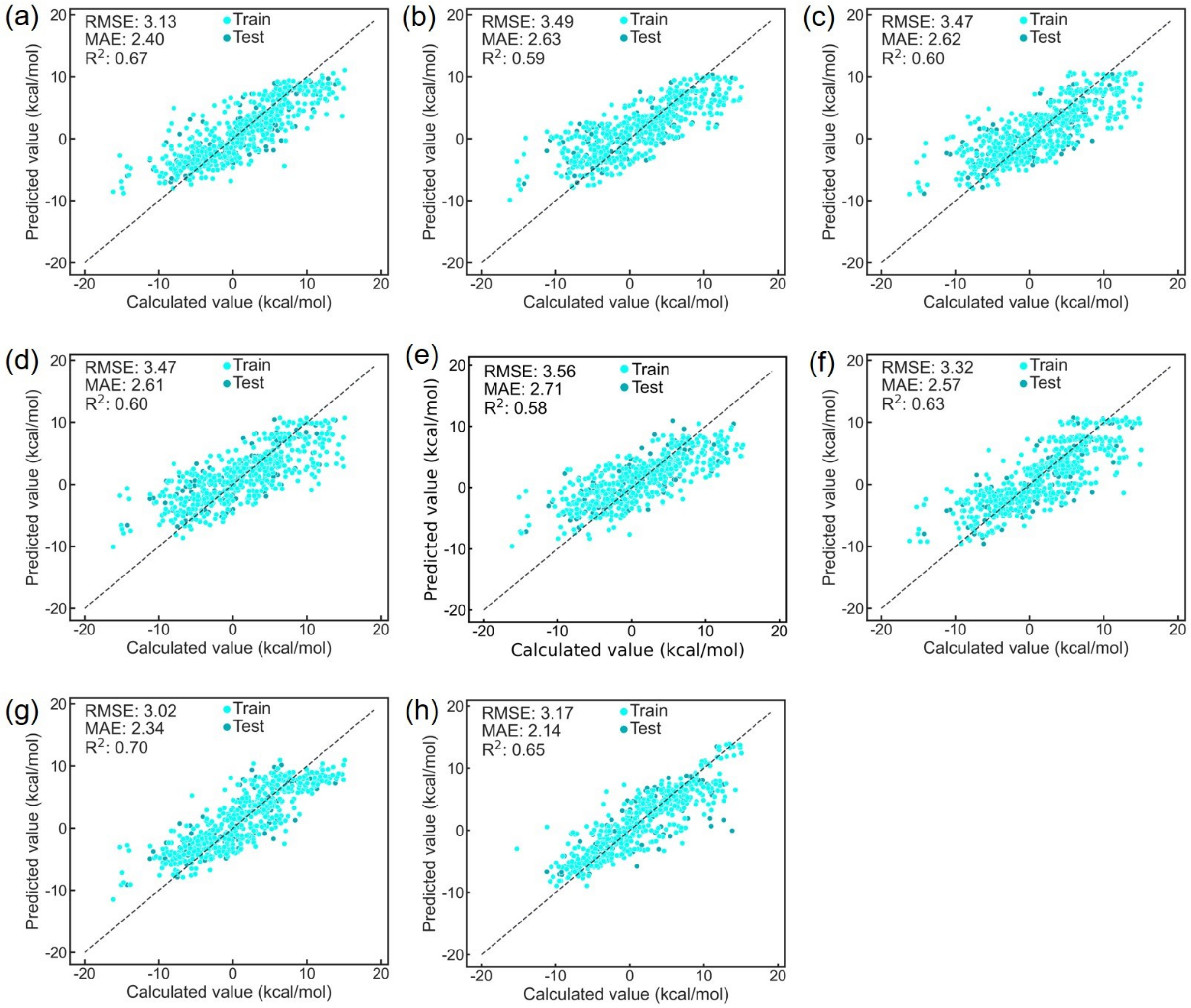

Figure S9. The plot of predicted versus calculated ΔE^* of (a) AtomPair, (b) ECFP, (c) RDKit, (d) Topological torsion, (e) Quantitative descriptor, (f) MACCS, (g) DMPNN, (h) Feature engineering descriptors used KNN model in CV at one of folds.

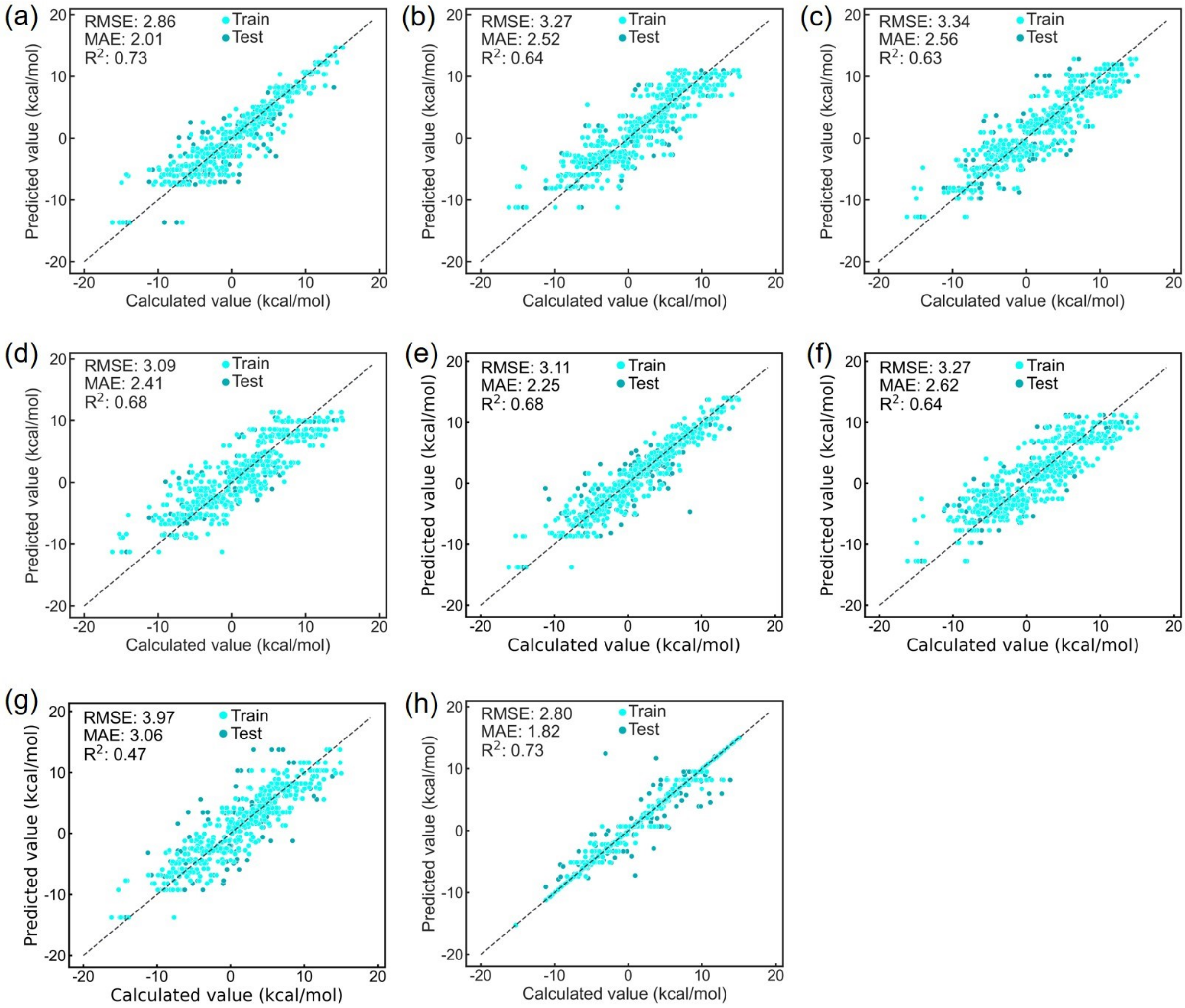

Figure S10. The plot of predicted versus calculated ΔE^* of (a) AtomPair, (b) ECFP, (c) RDKit, (d) Topological torsion, (e) Quantitative descriptor, (f) MACCS, (g) DMPNN, (h) Feature engineering descriptors used DT model in CV at one of folds.

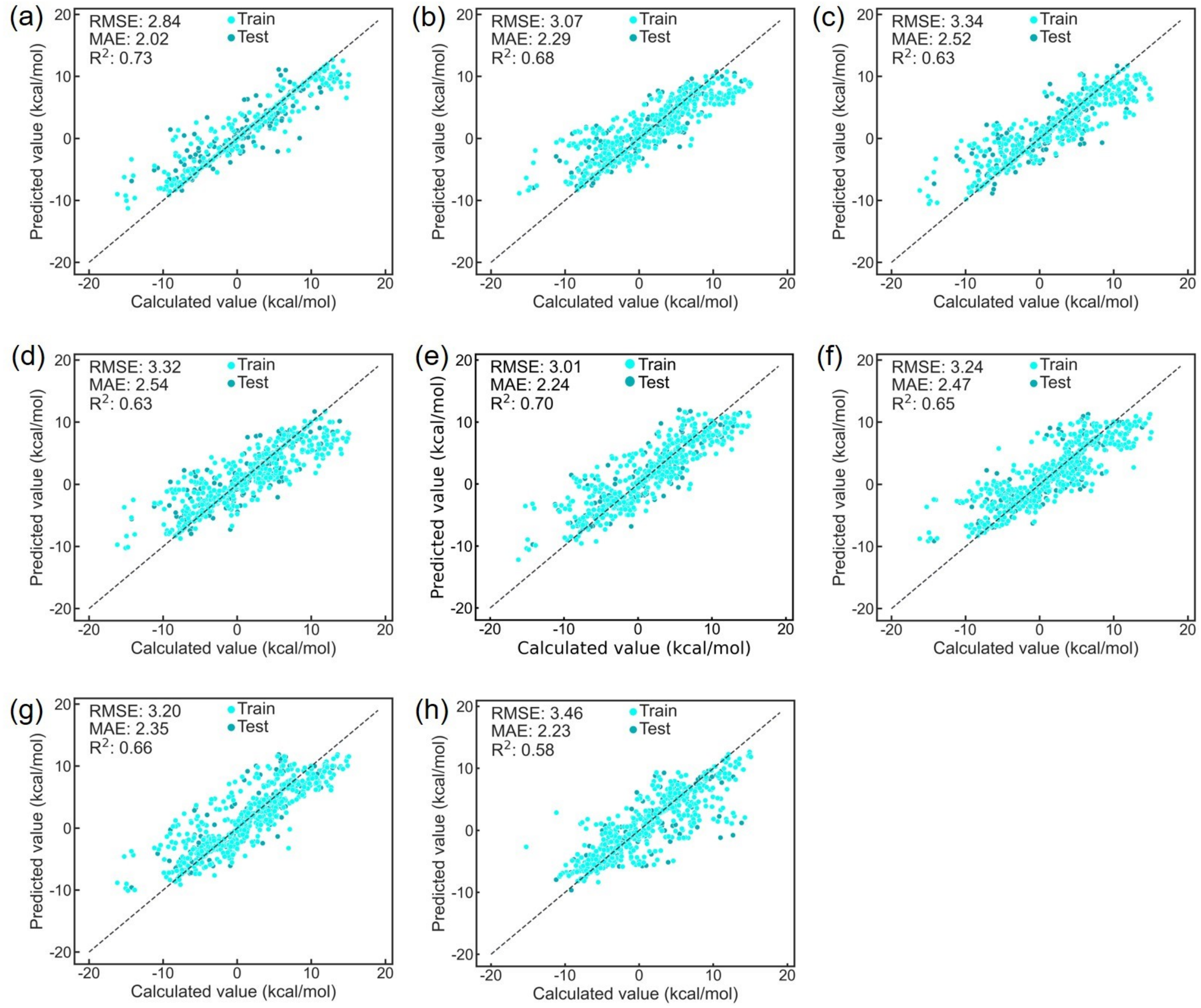

Figure S11. The plot of predicted versus calculated ΔE^* of (a) AtomPair, (b) ECFP, (c) RDKit, (d) Topological torsion, (e) Quantitative descriptor, (f) MACCS, (g) DMPNN, (h) Feature engineering descriptors used SVM model in CV at one of folds.

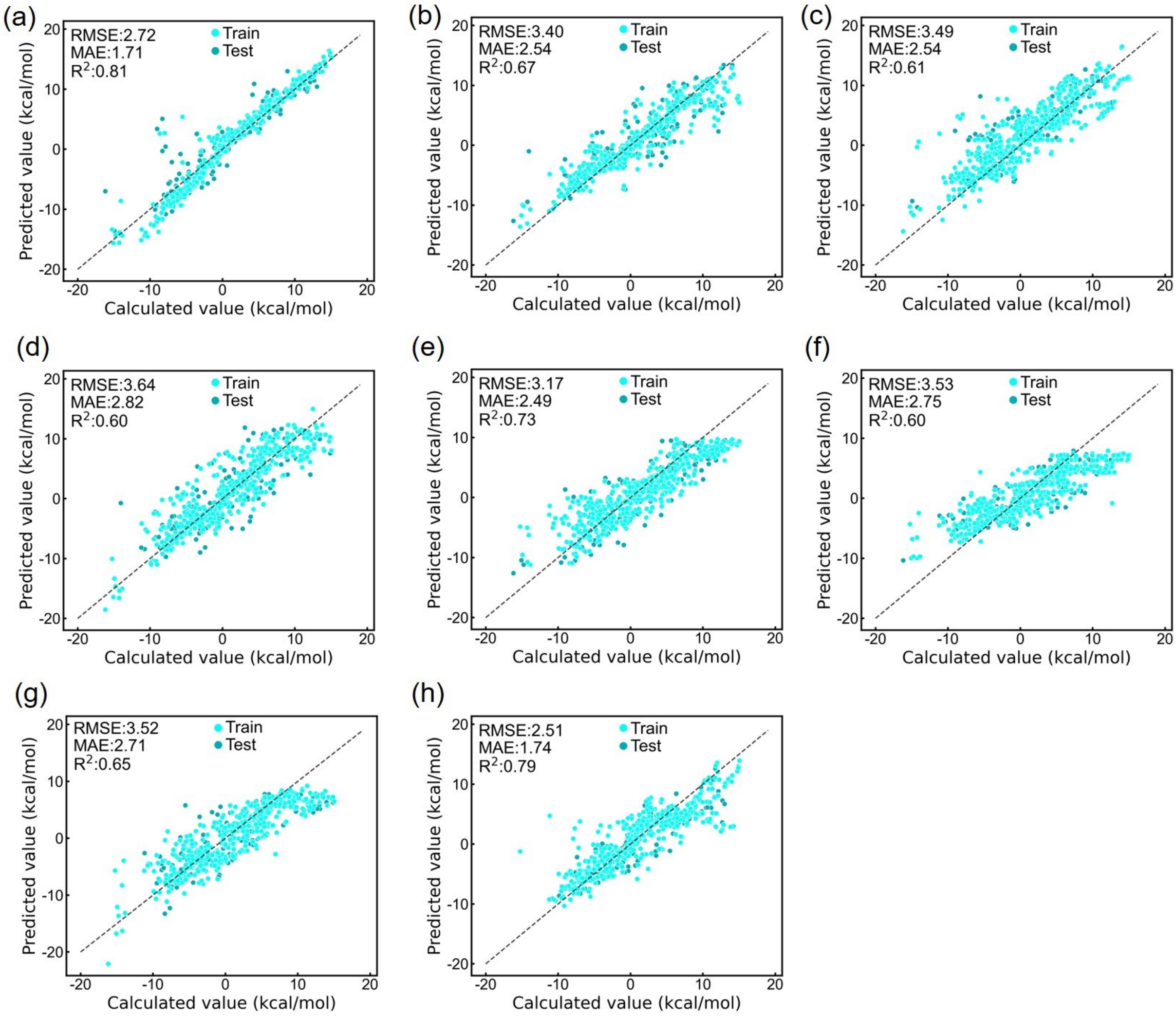

Figure S12. The plot of predicted versus calculated ΔE^* of (a) AtomPair, (b) ECFP, (c) RDKit, (d) Topological torsion, (e) Quantitative descriptor, (f) MACCS, (g) DMPNN, (h) Feature engineering descriptors used MLP model in CV at one of fold.

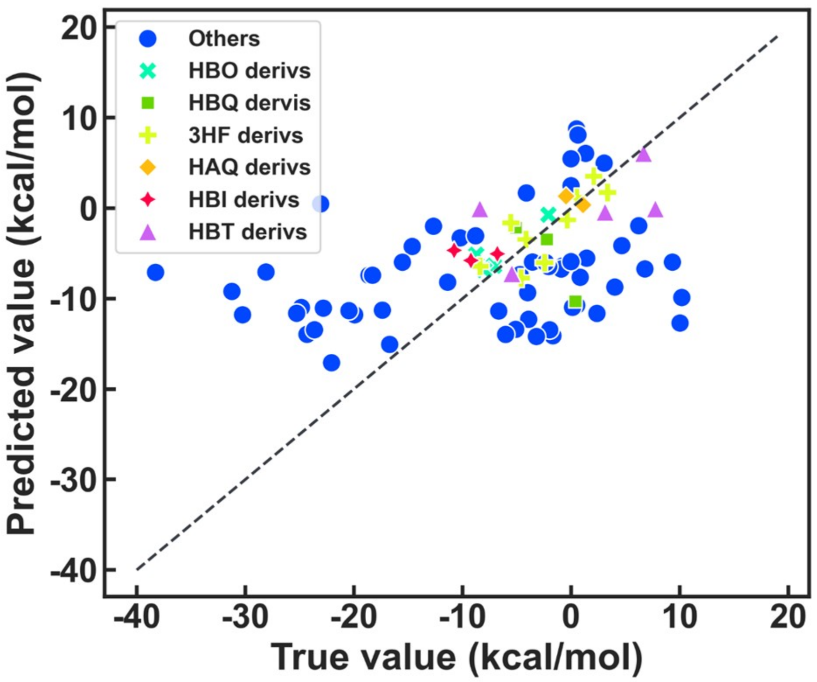

Figure S13. The plot of predicted versus calculated value of XGBoost model used atom-pair fingerprint as input on documented ESIPT molecules which the category "others" refers to molecules whose parent scaffolds are not included in the ESIPT dataset.

Based on the observed predictive performance of the model, reliable application is considered most reliable when the Δ*E** prediction model is applied to ESIPT molecules whose parent scaffolds fall within the six types contained in the dataset and whose estimated Δ*E** values lie within the training interval (−16 to 16 kcal/mol). Application beyond this scope, particularly for scaffolds outside the six represented types, is expected to result in significantly reduced prediction accuracy.

2. Graph-Based Models

The prediction results of the graph-based models were obtained using the DeepChem toolkit. Three graph convolution featurizers, namely MolGraphConvFeaturizer, ConvMolFeaturizer, and PagtnMolGraphFeaturizer, were employed to represent molecular structures. The MolGraphConvFeaturizer served as the input for the AttentiveFPModel, GATModel, and GCNModel. The ConvMolFeaturizer was used as the input for the GraphConvModel. The PagtnMolGraphFeaturizer was applied as an input for a variant of the PagtnModel. Compared to traditional strong ML algorithms, XGboost, RF, and GB, the graph-based models exhibited slightly lower predictive performance.

S1.3 Molecular Fingerprint

1. RDKit Fingerprints

This is an RDKit-specific fingerprint that is inspired by (though it differs significantly from) public descriptions of the Daylight fingerprint. The fingerprinting algorithm identifies all subgraphs in the molecule within a particular range of sizes, hashes each subgraph to generate a raw bit ID, mods that raw bit ID to fit in the assigned fingerprint size, and then sets the corresponding bit. Options are available to generate count-based forms of the fingerprint or "non-folded" forms (using a sparse representation). The default scheme for hashing subgraphs is to hash the individual bonds based on: (1) the types of the two atoms. Atom types include the atomic number (mod 128), and whether or not the atom is aromatic. (2) the degrees of the two atoms in the path. (3) the bond type (or AROMATIC if the bond is marked as aromatic).

2. Atom-Pair and Topological Torsion Fingerprints[4].

Atom-Pair and Topological Torsion fingerprints are two widely used molecular descriptors that capture structural features based on topological relationships between atoms. The Atom-Pair fingerprint represents a molecule by encoding all pairs of atoms along with their respective atom types and the shortest topological distance between them. This approach

captures both atomic identity and molecular shape, allowing for the quantification of molecular similarity. In contrast, the Topological Torsion fingerprint encodes linear sequences of four connected atoms (torsions), reflecting not only atom and bond types but also their sequential arrangement in the molecular graph. This method captures more local and sequential structural information compared to atom pairs, making it particularly useful in analyzing conformationally relevant substructures. Both fingerprints are used extensively in cheminformatics for tasks such as virtual screening, similarity searching, and QSAR modeling, and they offer complementary perspectives on molecular structure.

These fingerprint types are different from the others in the RDKit in that bits in the sparse form of the fingerprint can be directly explained (i.e. the "hashing function" used is fully reversible). These fingerprints were originally "intended" to be used in count-vectors and they seem to work better that way. The default behavior of the explicit bit-vector forms of both fingerprints is to use a "count simulation" procedure where multiple bits are set for a given feature if it occurs more than once. The default behavior is to use 4 fingerprint bits for each feature (so a 2048 bit fingerprint actually stores information about the same number of features as a 512 bit fingerprint that isn't using count simulation). The bins correspond to counts of 1, 2, 4, and 8. As an example of how this works: if a feature occurs 5 times in a molecule, the bits corresponding to counts 1, 2, and 4 will be set.

Representation of atom pair contained in a molecule: A·Xn-(n)-B·Xn. A and B represent atom names (such as C, O, N, etc). A dot ("·") following an atom name indicates the presence of a bonding π electron. The suffix Xn following an atom name indicates the presence of n non-hydrogen neighboring atoms. "-(n)-" in the middle represents the numbers of atoms in the atom pair (including the two atoms of atom pair).

3. ECFP

The extended connectivity fingerprint (ECFP) is a molecular fingerprint that can represent the internal structure of a compound, which is derived from the Morgan algorithm. In recent years, ECFP has become the industry standard method for circular molecular fingerprint, which

is often used as a benchmark to compare the effect of new methods in machine learning. Morgan circular fingerprint (MCP) is part of the family of ECFP, using Morgan generation algorithm. MCP searches substructures of all given steps in the compound through Morgan search algorithm, and then obtains the hash value of each substructure through hash, thus forming the corresponding fingerprint. When this fingerprint is used, it will produce a fingerprint of variable length according to different set diameters. They record each environment from the atom up to a specified radius. MCP can determine the absence or presence of molecular function and is widely used in similarity search of complete structures.

4. Quantitative Descriptors

Quantitative descriptors are numerical values that encode various physicochemical, structural, or electronic properties of molecules, enabling their use in machine learning models and quantitative structure–activity relationship (QSAR) studies. These descriptors capture essential molecular characteristics such as molecular weight, hydrophobicity (logP), polar surface area, partial charges, topological indices, and electronic properties derived from quantum chemical calculations. By converting complex molecular structures into a standardized numerical format, quantitative descriptors allow for systematic comparison, prediction of molecular behavior, and efficient modeling of structure–property or structure–activity relationships. Their interpretability and versatility make them fundamental tools in computational chemistry, drug discovery, and material design. Quantitative descriptors can be divided into 1D, 2D, and 3D, etc. according to the calculation of molecular structure dimensions required. RDKit provides many methods for calculating descriptors, which can be used for molecular screening, drugenicity assessment, etc. The quantitative descriptors used in our paper, which can be found in https://www.rdkit.org/docs/GettingStartedInPython.html#list-of-available-descriptors are listed below.

MolWt	Molecular Weight
HeavyAtomMolWt	Molecular weight of non-hydrogen atoms

ExactMolWt	Exact Molecular Weight
NumValenceElectrons	Number of valence electrons
NumRadicalElectrons	Number of radical electrons
MaxEStateIndex	Maximum E-State index
MinEStateIndex	Minimum E-State index
MaxAbsEStateIndex	Maximum absolute E-State index
MinAbsEStateIndex	Minimum absolute E-State index
NumHDonors	Number of H-bond donors
NumHAcceptors	Number of H-bond acceptors
NumRotatableBonds	Number of rotatable bonds
NumAromaticRings	Number of aromatic rings
NumAliphaticRings	Number of aliphatic rings
NumSaturatedRings	Number of saturated rings
NumHeteroaromaticRings	Number of heteroaromatic rings
NumAliphaticHeterocycles	Number of aliphatic heterocycles
NumSaturatedHeterocycles	Number of saturated heterocycles
NumAromaticCarbocycles	Number of aromatic carbocycles
NumAliphaticCarbocycles	Number of aliphatic carbocycles
NumSaturatedCarbocycles	Number of saturated carbocycles
RingCount	Total number of rings
MolLogP	Octanol-water partition coefficient (LogP)
MolMR	Molar Refractivity
TPSA	Topological Polar Surface Area
Chi0n	Kier & Hall Chi index (order 0)
Chi1n	Kier & Hall Chi index (order 1)
Chi2n	Kier & Hall Chi index (order 2)
Chi3n	Kier & Hall Chi index (order 3)

Chi4n	Kier & Hall Chi index (order 4)
Chi0v	Kier & Hall Valence Chi index (order 0)
Chi1v	Kier & Hall Valence Chi index (order 1)
Chi2v	Kier & Hall Valence Chi index (order 2)
Chi3v	Kier & Hall Valence Chi index (order 3)
Chi4v	Kier & Hall Valence Chi index (order 4)
BalabanJ	Balaban J connectivity index
BertzCT	Bertz complete topological index
Ipc	Information Content index
Kappa1	Hall-Kier Kappa shape index (order 1)
Kappa2	Hall-Kier Kappa shape index (order 2)
Kappa3	Hall-Kier Kappa shape index (order 3)
Phi	Sum of reciprocal square root of bond lengths
LabuteASA	Labute Approximate Surface Area
PEOE_VSA1	PEOE VSA descriptor (bin 1)
PEOE_VSA2	PEOE VSA descriptor (bin 2)
PEOE_VSA3	PEOE VSA descriptor (bin 3)
PEOE_VSA4	PEOE VSA descriptor (bin 4)
PEOE_VSA5	PEOE VSA descriptor (bin 5)
PEOE_VSA6	PEOE VSA descriptor (bin 6)
PEOE_VSA7	PEOE VSA descriptor (bin 7)
PEOE_VSA8	PEOE VSA descriptor (bin 8)
PEOE_VSA9	PEOE VSA descriptor (bin 9)
PEOE_VSA10	PEOE VSA descriptor (bin 10)
PEOE_VSA11	PEOE VSA descriptor (bin 11)
PEOE_VSA12	PEOE VSA descriptor (bin 12)
PEOE_VSA13	PEOE VSA descriptor (bin 13)

PEOE_VSA14	PEOE VSA descriptor (bin 14)
SMR_VSA1	Molar Refractivity VSA descriptor (bin 1)
SMR_VSA2	Molar Refractivity VSA descriptor (bin 2)
SMR_VSA3	Molar Refractivity VSA descriptor (bin 3)
SMR_VSA4	Molar Refractivity VSA descriptor (bin 4)
SMR_VSA5	Molar Refractivity VSA descriptor (bin 5)
SMR_VSA6	Molar Refractivity VSA descriptor (bin 6)
SMR_VSA7	Molar Refractivity VSA descriptor (bin 7)
SMR_VSA8	Molar Refractivity VSA descriptor (bin 8)
SMR_VSA9	Molar Refractivity VSA descriptor (bin 9)
SMR_VSA10	Molar Refractivity VSA descriptor (bin 10)
SMR_VSA11	Molar Refractivity VSA descriptor (bin 11)
SMR_VSA12	Molar Refractivity VSA descriptor (bin 12)
EState_VSA1	E-State VSA descriptor (bin 1)
EState_VSA2	E-State VSA descriptor (bin 2)
EState_VSA3	E-State VSA descriptor (bin 3)
EState_VSA4	E-State VSA descriptor (bin 4)
EState_VSA5	E-State VSA descriptor (bin 5)
EState_VSA6	E-State VSA descriptor (bin 6)
EState_VSA7	E-State VSA descriptor (bin 7)
EState_VSA8	E-State VSA descriptor (bin 8)
EState_VSA9	E-State VSA descriptor (bin 9)
EState_VSA10	E-State VSA descriptor (bin 10)
EState_VSA11	E-State VSA descriptor (bin 11)
VSA_EState1	VSA E-State descriptor (bin 1)
VSA_EState2	VSA E-State descriptor (bin 2)
VSA_EState3	VSA E-State descriptor (bin 3)

VSA_EState4	VSA E-State descriptor (bin 4)
VSA_EState5	VSA E-State descriptor (bin 5)
VSA_EState6	VSA E-State descriptor (bin 6)
VSA_EState7	VSA E-State descriptor (bin 7)
VSA_EState8	VSA E-State descriptor (bin 8)
VSA_EState9	VSA E-State descriptor (bin 9)
VSA_EState10	VSA E-State descriptor (bin 10)
FractionCSP3	Fraction of sp3 hybridized carbons
NumAlcohols	Number of alcohol groups
NumPrimaryAlcohols	Number of primary alcohols (C-OH)
NumSecondaryAlcohols	Number of secondary alcohols (C2CH-OH)
NumTertAlcohols	Number of tertiary alcohols (C3C-OH)
NumAmides	Number of amide groups
NumPrimaryAmides	Number of primary amides (CONH2)
NumSecondaryAmides	Number of secondary amides (CONHCR)
NumTertAmides	Number of tertiary amides (CONR2)
NumAmines	Number of amine groups
NumPrimaryAmines	Number of primary amines (NH2)
NumSecondaryAmines	Number of secondary amines (NHR)
NumTertAmines	Number of tertiary amines (NR3)
NumCarboxilicAcids	Number of carboxylic acid groups
NumEsters	Number of ester groups
NumEthers	Number of ether groups
NumHalides	Number of halide atoms (F Cl Br l)
NumHeteroatoms	Number of heteroatoms (B, N, O, F, Si, P, S, Cl, As, Se, Br, Te, I, At)
NumLipinskiHBA	Lipinski H-bond acceptors (<= 10)

NumLipinskiHBD	Lipinski H-bond donors (<= 5)
NumAromaticHeterocycles	Number of aromatic heterocycles
FpDensityMorgan1	Morgan Fingerprint Density (Radius 1)
FpDensityMorgan2	Morgan Fingerprint Density (Radius 2)
FpDensityMorgan3	Morgan Fingerprint Density (Radius 3)
BCUT2D_MWHI	Burden Eigendensity (MW high)
BCUT2D_MWLOW	Burden Eigendensity (MW low)
BCUT2D_CHGHI	Burden Eigendensity (Charge high)
BCUT2D_CHGLOW	Burden Eigendensity (Charge low)
BCUT2D_HOMO	Burden Eigendensity (HOMO-like high)
BCUT2D_LUMO	Burden Eigendensity (LUMO-like low)
BCUT2D_MRHI	Burden Eigendensity (MR high)
BCUT2D_MRLOW	Burden Eigendensity (MR low)
MinAbsPartialCharge	Minimum absolute partial charge
MaxAbsPartialCharge	Maximum absolute partial charge
MinPartialCharge	Minimum partial charge
MaxPartialCharge	Maximum partial charge
AvgInterBondDistance	Average inter-bond distance
AvgInterBondVector	Average inter-bond vector
AvgInterBondAngle	Average inter-bond angle
NumBonds	Total number of bonds
NumBridgeheadAtoms	Number of bridgehead atoms
NumSpiroAtoms	Number of spiro atoms
NumAtoms	Total number of atoms (including H)
HeavyAtomCount	Number of heavy (non-hydrogen) atoms
NumH	Number of hydrogen atoms
fr_Al_COO	Count of aliphatic COO (carboxylic acid or ester)

fr_Al_OH	Count of aliphatic alcohols (C-OH)
fr_Al_OH_noTert	Count of aliphatic alcohols excluding tertiary
fr_Ar_N	Count of aromatic N
fr_Ar_NH	Count of aromatic NH
fr_Ar_OH	Count of aromatic OH (phenols)
fr_BnzlC	Count of benzyl carbons
fr_C_O	Count of C=O (excluding acid ester amide)
fr_C_O_noCOO	Count of C=O (excluding acid ester)
fr_C_S	Count of C-S bonds (excluding thiols)
fr_HOCCN	Count of N attached to C=O or S=O or P=O (amide-like)
fr_Imine	Count of imine groups (C=N)
fr_NH0	Count of tertiary amines/amides (NR3 or CONR2)
fr_NH1	Count of secondary amines/amides (NHR or CONHR)
fr_NH2	Count of primary amines/amides (NH2 or CONH2)
fr_N_O	Count of N-O bonds
fr_Ndealkylation1	Count of N dealkylation sites (type 1)
fr_Ndealkylation2	Count of N dealkylation sites (type 2)
fr_Nhpyrrole	Count of non-hydrogenic pyrrole N
fr_SH	Count of thiol groups (SH)
fr_aldehyde	Count of aldehyde groups (CHO)
fr_alkyl_carbamate	Count of alkyl carbamates
fr_alkyl_halide	Count of alkyl halides (C-Hal)
fr_allylic_oxid	Count of allylic oxidation sites (C=C-C-H)
fr_amide	Count of non-acid amides
fr_amidine	Count of amidine groups (C(=N)N)
fr_aniline	Count of aniline-like groups (C6H5N)
fr_aryl_methyl	Count of C6H5CH3 groups

fr_azide	Count of azide groups (N3)
fr_azo	Count of azo groups (N=N)
fr_barbitur	Count of barbiturate groups
fr_bicyclic	Count of bicyclic systems
fr_ester	Count of ester groups
fr_ether	Count of ether groups
fr_guanido	Count of guanidine groups (N=C(N)N)
fr_halogen	Count of halogens (F Cl Br I)
fr_hdrzine	Count of hydrazine groups (N-N)
fr_hdrzone	Count of hydrazone groups (C=N-N)
fr_imidazole	Count of imidazole rings
fr_isocyan	Count of isocyanate groups (N=C=O)
fr_isothiocyan	Count of isothiocyanate groups (N=C=S)
fr_ketone	Count of ketones (C=O, excluding acid, ester, amide)
fr_ketone_Topliss	Count of ketones for Topliss scheme
fr_lactam	Count of lactam rings (cyclic amides)
fr_lactone	Count of lactone rings (cyclic esters)
fr_methoxy	Count of methoxy groups (OCH3)
fr_oxime	Count of oxime groups (C=NOH)
fr_para_hydroxylation	Count of para hydroxylation sites on benzene
fr_phenol	Count of phenol groups (Ar-OH)
fr_phenol_noOrthoH	Count of phenols without ortho H
fr_phos_acid	Count of phosphoric acid groups (P(=O)(OH)2)
fr_phos_ester	Count of phosphoric ester groups
fr_piperdine	Count of piperdine rings (saturated N-heterocycle)
fr_priamide	Count of primary amides (CONH2)
fr_quatN	Count of quaternary nitrogen atoms (N+)

fr_sulfide	Count of sulfide groups (C-S-C)
fr_sulfonamd	Count of sulfonamide groups (SO2N)
fr_sulfone	Count of sulfone groups (SO2)
fr_term_acetylene	Count of terminal acetylene (C=CH)
fr_tetrazole	Count of tetrazole rings (N4C)
fr_thiazole	Count of thiazole rings
fr_thiocyan	Count of thiocyanate groups (S-C=N)
fr_thiophene	Count of thiophene rings
fr_unbrch_alkane	Count of unbranched alkane chains (C4 to C7)
fr_urea	Count of urea groups (NCON)

5. MACCS

The MACCS fingerprints[5] (Molecular ACCess System keys) is a widely used structural representation method in cheminformatics, particularly effective for tasks such as molecular similarity analysis, virtual screening, and quantitative structure–activity relationship (QSAR) modeling. It consists of a predefined set of structural keys, with the most common version comprising 166 binary bits. Each bit corresponds to the presence or absence of a specific substructure or functional group within a molecule, such as alkyl chains, aromatic rings, esters, or amines. A value of 1 indicates the presence of the corresponding structural feature, while 0 indicates its absence. Due to its fixed length and well-defined structural interpretation, the MACCS fingerprint offers computational efficiency and interpretability. However, it is relatively limited in capturing complex molecular topologies or long-range interactions compared to more sophisticated fingerprints like ECFP. Despite this, MACCS fingerprints remain a popular and practical tool in various cheminformatics applications. The fragment definitions for the MACCS 166 keys can be found in this document: https://github.com/rdkit/rdkit/blob/master/rdkit/Chem/MACCSkeys.py. The list of MACCS 166 keys is following.

Index	SMARTS Pattern	Description
1	?	Isotope (generic wildcard for any atom)
2	[#104]	Element with atomic number 104 (Rutherfordium)
3	[#32,#33,#34,# 50,#51,#52,#82,#83,#84]	Group IVa, Va, VIa elements (Si, P, S, Ge, As, Se, Te, Po)
4	[Ac,Th,Pa,U,Np,Pu,Am,Cm,Bk, Cf,Es,Fm,Md,No,Lr]	Actinide elements
5	[Sc,Ti,Y,Zr,Hf]	Group IIIB, IVB elements (Scandium, Titanium, Yttrium, Zirconium, Hafnium)
6	[La,Ce,Pr,Nd,P m,Sm,Eu,Gd,Tb ,Dy,Ho,Er,Tm,Yb,Lu]	Lanthanide elements
7	[V,Cr,Mn,Nb,Mo,Tc,Ta,W,Re]	Group VB, VIB, VIIB elements (Vanadium, Chromium, Manganese, etc.)
8	[!#6;!#1]1~*~*~*~1	Aromatic ring with specific conditions (carbon atoms and hydrogens excluded)
9	[Fe,Co,Ni,Ru,Rh,Pd,Os,Ir,Pt]	Group VIII transition metals (Fe, Co, Ni, etc.)
10	[Be,Mg,Ca,Sr,Ba,Ra]	Group IIa (Alkaline Earth metals)
11	*1~*~*~*~1	4-membered ring structure
12	[Cu,Zn,Ag,Cd,Au,Hg]	Group IB, IIB (Cu, Zn, Ag, etc.)
13	[#8]~[#7](~[#6])~[#6]	A structure involving oxygen, nitrogen, and carbon atoms
14	[#16]-[#16]	Disulfide bond (S-S)
15	[#8]~[#6](~[#8])~[#8]	Structure involving oxygen and carbon atoms
16	[!#6;!#1]1~*~*~1	Aromatic ring structure excluding carbon and hydrogen

17	[#6]#[#6]	Carbon-carbon triple bond (C≡C)
18	[#5,#13,#31,#49,#81]	Group IIIA elements (Boron, Aluminum, Gallium, Indium, Thallium)
19	*1~*~*~*~*~*~*~1	7-membered ring structure
20	[#14]	Silicon (Si)
21	[#6]=[#6](~[!#6; !#1])~[!#6;!#1]	Vinyl structure with specific substitutions
22	*1~*~*~1	3-membered ring structure
23	[#7]~[#6](~[#8])~[#8]	Nitrogen-carbon-oxygen structure
24	[#7]-[#8]	Nitrogen-oxygen single bond
25	[#7]~[#6](~[#7])~[#7]	Nitrogen-carbon-nitrogen structure
26	[#6]=;@[#6](@*)@*	C=C structure with additional groups
27	[I]	Iodine atom
28	[!#6;!#1]~[CH2]~[!#6;!#1]	Structure with a CH2 group between non-carbon and non-hydrogen atoms
29	[#15]	Phosphorus (P)
30	[#6]~[!#6;!#1](~[#6])(~[#6])~*	Carbon structure with specific substitutions and functional groups
31	[!#6;!#1]~[F,Cl,Br,I]	Halogen atoms (F, Cl, Br, I)
32	[#6]~[#16]~[#7]	Carbon-sulfur-nitrogen structure
33	[#7]~[#16]	Nitrogen-sulfur bond
34	[CH2]=*	C-H group with double bond to another atom
35	[Li,Na,K,Rb,Cs,Fr]	Group IA (Alkali metals: Li, Na, K, etc.)
36	[#16R]	Sulfur in a heterocycle (part of a ring)
37	[#7]~[#6](~[#8])~[#7]	Nitrogen-carbon-oxygen-nitrogen structure
38	[#7]~[#6](~[#6])~[#7]	Nitrogen-carbon-carbon-nitrogen structure
39	[#8]~[#16](~[#8])~[#8]	Oxygen-sulfur-oxygen structure

40	[#16]-[#8]	Sulfur-oxygen single bond (S-O)
41	[#6]#[#7]	Carbon-nitrogen triple bond (C≡N)
42	F	Fluorine atom
43	[!#6;!#1;!H0]~ *~[!#6;!#1;!H0]	Structure with no hydrogens and non-c arbon and non-hydrogen atoms
44	[!#1;!#6;!#7;!#8;!# 9;!#14;!#15;!#16;!# 17;!#35;!#53]	Non-hydrogen, non-carbon, non-h eteroatom (others)
45	[#6]=[#6]~[#7]	C=C-N structure
46	Br	Bromine atom
47	[#16]~*~[#7]	Sulfur-nitrogen structure
48	[#8]~[!#6;!#1](~ [#8])(~[#8])	Oxygen with two additional oxygen atoms
49	[!+0]	Charge (generic)
50	[#6]=[#6](~[#6])~[#6]	C=C(C)C structure
51	[#6]~[#16]~[#8]	Carbon-sulfur-oxygen structure
52	[#7]~[#7]	Nitrogen-nitrogen single bond
53	[!#6;!#1;!H0]~*~*~* ~[!#6;!#1;!H0]	Structure with no hydrogens and non-carbon and non-hydrogen atoms
54	[!#6;!#1;!H0]~*~*~[!#6;!#1;!H0]	Structure with no hydrogens and non-carbon and non-hydrogen atoms
55	[#8]~[#16]~[#8]	Oxygen-sulfur-oxygen structure
56	[#8]~[#7](~[#8])~[#6]	Oxygen-nitrogen-carbon structure
57	[#8R]	Oxygen in a heterocycle (part of a ring)
58	[!#6;!#1]~[#16]~[!#6;!#1]	Structure with sulfur between non-carbon and non-hydrogen atoms
59	[#16]!:*:*	Sulfur with additional atoms (not specified)
60	[#16]~*~[#16]	Sulfur-sulfur bond (S-S)

61	[!#6;!#1;!#16]~*~[#6]	Structure with sulfur between non-carbon and carbon atoms
62	[!#6]~[#16]~[#6]	Sulfur-carbon bond
63	[!#6]~[#6]~[#16]	Carbon-sulfur structure with specified bonding
64	[!#1;!#6]~[#16]~[!#6;!#1]	Structure with sulfur between non-carbon and non-hydrogen atoms
65	[!#1;!#6]~[!#16]~[#7]	Non-hydrogen, non-carbon with nitrogen
66	[!#7]~[#8]~[#7]	Nitrogen-oxygen-nitrogen str ucture
67	[!#6;!#1]~*~[#6]~[#7]	Non-carbon, non-hydrogen st ructure with carbon-nitrogen bonding
68	[!#1]~[CH2]~[!#1]	Structure with hydrogen-free CH2 group
69	[#8]~[!#6;!#1]~[CH2]	Structure involving oxygen and a CH2 group
70	[!#6]~[#8]~[#7]~[!#8]	Structure with oxygen and nitrogen atoms without a third oxygen
71	[!#1]~[Cl,Br]~[#16]~[*]	Halogen, sulfur bond structure
72	[!#6]~[C,N,O,S]~[!#1]~[*]	Structure with carbon, nitrogen, oxygen, s ulfur, and non-hydrogen atoms
73	[!#1]~[N]~[*]	Structure involving nitrogen with an arbitrary atom
74	[!#6]~[#16]~[!#8]~[*]	Sulfur-carbon structure involving non-oxygen atoms
75	[!#6]~[#6]~[!#1]~[*]	Carbon-carbon bonding structure excluding hydrogens
76	[*]~[#8]~[Cl]~[#6]	Oxygen-hydrogen-chlorine-carbon bonding
77	[C,N,O]~[*]~[*]	Carbon, nitrogen, or oxygen bonded with any atom

78	[!#6]~[#7]~[#16]~[*]	Nitrogen-sulfur structure with a free atom
79	[F]~[#16]~[#8]~[*]	Fluorine-sulfur-oxygen structure
80	[O]~[*]~[*]	Oxygen with unspecified other atom
81	[!#1]~[*]~[#7]~[O]	Nitrogen-oxygen bonded structure
82	[S]~[*]~[C]~[Cl]	Sulfur-carbon-chlorine structure
83	[C,O]~[*]~[*]~[#7]	Carbon-oxygen-nitrogen bonded structure
84	[NH2]	Amine group (NH2)
85	[#6]~[#7](~[#6])~[#6]	Carbon-nitrogen-carbon-carbon structure
86	[C;H2,H3][!#6;!#1] [C;H2,H3]	Hydrocarbon bridge between two non-carbon atoms
87	[F,Cl,Br,I]!@*@*	Halogen atom bonding to arbitrary atom
88	[#16]	Sulfur atom
89	[#8]~*~*~*~[#8]	Oxygen atom with surrounding structure
90	[$([!#6;!#1;!H0] ~*~*~[CH2]~*), $([!#6;!#1;!H0 ;R]1@[R]@[R]@[CH2;R]1), $([!#6;!#1;!H0]~[R]1@[R]@[C H2;R]1)]	Specific structure with CH2 bridge and exclusion of hydrogens
91	[$([!#6;!#1;!H0] ~*~*~*~[CH2]~*), $([!#6;!#1;!H0; R]1@[R]@[R] @[R]@[CH2;R]1), $([!#6;!#1;!H0]~[R]1@[R]@[R]@[C H2;R]1), $([!#6;!#1;!H0]~	Extended version of th e CH2-containing structure

	*~[R]1@[R]@[CH2;R]1)]	
92	[#8]~[#6](~[#7])~[#6]	Oxygen-carbon-nitrogen-carbon structure
93	[!#6;!#1]~[CH3]	Non-carbon, non-hydrogen with CH3 group
94	[!#6;!#1]~[#7]	Non-carbon, non-hydrogen with nitrogen
95	[#7]~*~*~[#8]	Nitrogen bonding to oxygen (N-O)
96	*1~*~*~*~*~*~1	5-membered ring structure
97	[#7]~*~*~*~[#8]	Nitrogen-oxygen structure (NAAO)
98	[!#6;!#1]1~*~*~*~*~*~1	Aromatic ring structure excluding specific atoms
99	[#6]=[#6]	Double bond between two carbon atoms (C=C)
100	*~[CH2]~[#7]	Hydrocarbon group (CH2) bonded to nitrogen (C-H-N)
101	[$([R]@1@[R]@[R]@[R]@[R]@[R]@ [R]@[R]1), $([R]@1@[R]@[R]@ [R]@[R]@[R] @[R]@[R]@[R]1), $([R]@1@[R]@[R] @[R]@[R]@[R] @[R]@[R]@[R]@[R]1), $([R]@1@[R]@[R] @[R]@[R]@[R] @[R]@[R]@[R]@ [R]@[R]@[R]1), $([R]@1@[R]@[R]@ [R]@[R]@[R]@[	Large ring structure (up to 14 members)

	R]@[R]@[R]@[R] @[R]@[R]@[R]1)	
102	[!#6;!#1]~[#8]	Non-carbon, non-hydrogen bonded to oxygen
103	Cl	Chlorine atom
104	[!#6;!#1;!H0]~*~[CH2]~*	Non-carbon, non-hydrogen bonded to CH2 group
105	*@*(@*)@*	A$A($A)$A structure
106	[!#6;!#1]~*(~[!#6;!#1])~[!#6;!#1]	QA(Q)Q structure
107	[F,Cl,Br,I]~*(~*)~*	Halogens bonding to arbitrary atoms
108	[CH3]~*~*~*~[CH2]~*	CH3-AA-CH2-A structure
109	*~[CH2]~[#8]	ACH2O structure
110	[#7]~[#6]~[#8]	Nitrogen-carbon-oxygen structure
111	[#7]~*~[CH2]~*	Nitrogen-CH2 bond structure
112	*~*(~*)(~*)~*	AA(A)(A)A structure
113	[#8]!:*:*	Onot%A%A structure
114	[CH3]~[CH2]~*	CH3CH2A structure
115	[CH3]~*~[CH2]~*	CH3ACH2A structure
116	[$([CH3]~*~*~[CH2]~*), $([CH3]~*1~*~[CH2]1)]	CH3AACH2A structure
117	[#7]~*~[#8]	Nitrogen-oxygen structure (NAO)
118	[$(*~[CH2]~[CH2]~*), $([R]1@[CH2;R]@ [CH2;R]1)]	ACH2CH2A structure
119	[#7]=*	Nitrogen double bond (N=A)
120	[!#6;R]	Heterocyclic atoms with additional conditions
121	[#7;R]	Nitrogen in heterocycle
122	*~[#7](~*)~*	AN(A)A structure

123	[#8]~[#6]~[#8]	Oxygen-carbon-oxygen structure
124	[!#6;!#1]~[!#6;!#1]	QQ structure (two identical atoms)
125	?	Aromatic Ring > 1
126	*!@[#8]!@*	A!O!A structure
127	*@*!@[#8]	A$A!O structure
128	[$(*~[CH2]~*~*~*~[CH2]~*), $([R]1@[CH2;R]@[R]@[R]@[R]@[CH2;R]1), $(*~[CH2]~[R]1@[R]@[R]@[CH2;R]1), $(*~[CH2]~*~[R]1@[R]@[CH2;R]1)]	ACH2AAACH2A structure
129	[$(*~[CH2]~*~*~[CH2]~*), $([R]1@[CH2]@[R]@[R]@[CH2;R]1), $(*~[CH2]~[R]1@[R]@[CH2;R]1)]	ACH2AACH2A structure
130	[!#6;!#1]~[!#6;!#1]	QQ > 1 (not fully specified)
131	[!#6;!#1;!H0]	QH > 1 (not fully specified)
132	[#8]~*~[CH2]~*	OACH2A structure
133	*@*!@[#7]	A$A!N structure
134	[F,Cl,Br,I]	Halogen group (F, Cl, Br, I)
135	[#7]!:*:*	Nnot%A%A structure
136	[#8]=*	Oxygen double bond with

		unspecified bonding (O=A)
137	[!C;!c;R]	Heterocycle
138	[!#6;!#1]~[CH2]~*	QCH2A > 1 (not fully specified)
139	[O;!H0]	Hydroxyl group (OH)
140	[#8]	Oxygen atom (O)
141	[CH3]	Methyl group (CH3)
142	[#7]	Nitrogen atom (N)
143	*@*!@[#8]	A$A!O structure
144	*!*:*:*!:*	Anot%A%Anot%A structure
145	*1~*~*~*~*~*~1	6-membered ring (aromatic or otherwise)
146	[#8]	Oxygen atom (O)
147	[$(*~[CH2]~[CH2]~*),<br>$([R]1@[CH2<br>;R]@[CH2;R]1)]	ACH2CH2A structure
148	*~[!#6;!#1](~*)~*	AQ(A)A structure
149	[C;H3,H4]	Methyl group (CH3) > 1 structure
150	*!@*@*!@*	A!A$A!A structure
151	[#7;!H0]	Nitrogen atom (NH)
152	[#8]~[#6](~[#6])~[#6]	Oxygen-carbon-carbon-carbon structure
153	[!#6;!#1]~[CH2]~*	QCH2A structure
154	[#6]=[#8]	Carbon-oxygen double bond (C=O)
155	*!@[CH2]!@*	A!CH2!A structure
156	[#7]~*(~*)~*	NA(A)A structure
157	[#6]-[#8]	Carbon-oxygen single bond (C-O)
158	[#6]-[#7]	Carbon-nitrogen single bond (C-N)
159	[#8]	Oxygen atom > 1
160	[C;H3,H4]	Methyl group (CH3)

161	[#7]	Nitrogen atom (N)
162	a	Aromatic atom
163	*1~*~*~*~*~*~1	6-membered aromatic ring structure
164	[#8]	Oxygen atom
165	[R]	Ring structure
166	?	Fragment structure (fix: this can't be done in SMARTS)

6. DMPNN

DMPNN[6] is a featurizer for Directed Message Passing Neural Network (D-MPNN) implementation. The default node representation are constructed by concatenating the following values, and the feature length is 133: Atomic num: A one-hot vector of this atom, in a range of first 100 atoms; Degree: A one-hot vector of the degree (0-5) of this atom; Formal charge: Integer electronic charge, -1, -2, 1, 2, 0; Chirality: A one-hot vector of the chirality tag (0-3) of this atom; Number of Hydrogens: A one-hot vector of the number of hydrogens (0-4) that this atom connected; Hybridization: A one-hot vector of "SP", "SP2", "SP3", "SP3D", "SP3D2"; Aromatic: A one-hot vector of whether the atom belongs to an aromatic ring; Mass: Atomic mass×0.01.

The default edge representation are constructed by concatenating the following values, and the feature length is 14: Bond type: A one-hot vector of the bond type, "single", "double", "triple", or "aromatic"; Same ring: A one-hot vector of whether the atoms in the pair are in the same ring; Conjugated: A one-hot vector of whether this bond is conjugated or not; Stereo: A one-hot vector of the stereo configuration (0-5) of a bond. The model's initial atom and bond features are provided below:[6]

feature	description	size
atom type	type of atom (ex. C, N, O), by atomic number	100
# bonds	number of bonds the atom is involved in	6

formal charge	integer electronic charge assigned to atom	5
chirality	unspecified, tetrahedral CW/CCW, or other	4
# Hs	number of bonded hydrogen atoms	5
hybridization	sp, sp2, sp3, sp3d, or sp3d2	5
aromaticity	whether this atom is part of an aromatic system	1
atomic mass	mass of the atom, divided by 100	1
bond type	single, double, triple, or aromatic	4
conjugated	whether the bond is conjugated	1
in ring	whether the bond is part of a ring	1
stereo	none, any, E/Z or cis/trans	6

S1.4 Clustering Analysis and Visualization

The feature representation of the ESIPT molecules was conducted using quantitative descriptors, qualitative descriptors (Atom-pair, RDkit, ECFP, Topotorsion descriptor) and molecular graphs from the RDKit and DeepChem toolkit, with each molecule using qualitative descriptors represented as a 2048-dimensional vector. Qualitative descriptors computed the representation of a molecule by decomposing it into local neighborhoods and hashing these components into a bit vector of the specified size, which can effectively describe the structural characteristics of molecules. The qualitative descriptors were used as input for clustering using the t-distributed stochastic neighbor embedding (tSNE) and Principal component analysis (PCA) algorithm in the scikit-learn toolkit. The clustering results were colored according to the category of molecules, with the HBO, HBQ, HAQ, 3HF, HBI and HBT derivatives corresponding to different colors respectively.

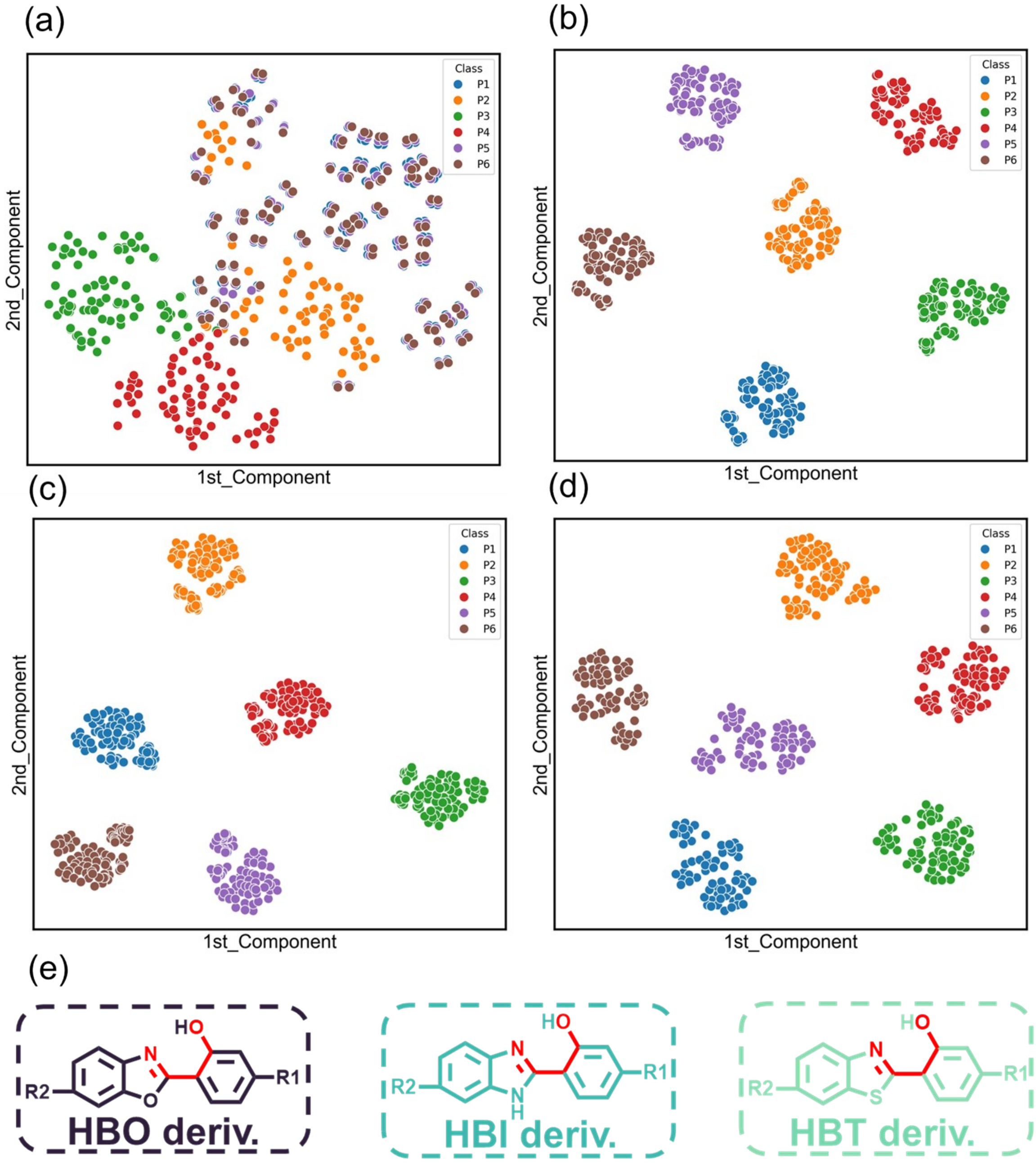

Figure S14. Visualization of the ESIPT molecular dataset based on t-SNE clustering method for (a) atom-pair, (b) RDKit, (c) ECFP, (d) topotorsion descriptor. Each point represents a molecule. P1, P2, P3, P4, P5, and P6 mean the derivatives of HBO, HBQ, 3HF, HAQ, HBI and HBT, respectively. The HBO, HBQ, 3HF, HAQ, HBI and HBT derivatives are marked with different colors respectively. (e) The NX2-(5)-OX1 atom pair (highlighted in red) in HBO, HBI, and HBT derivatives.

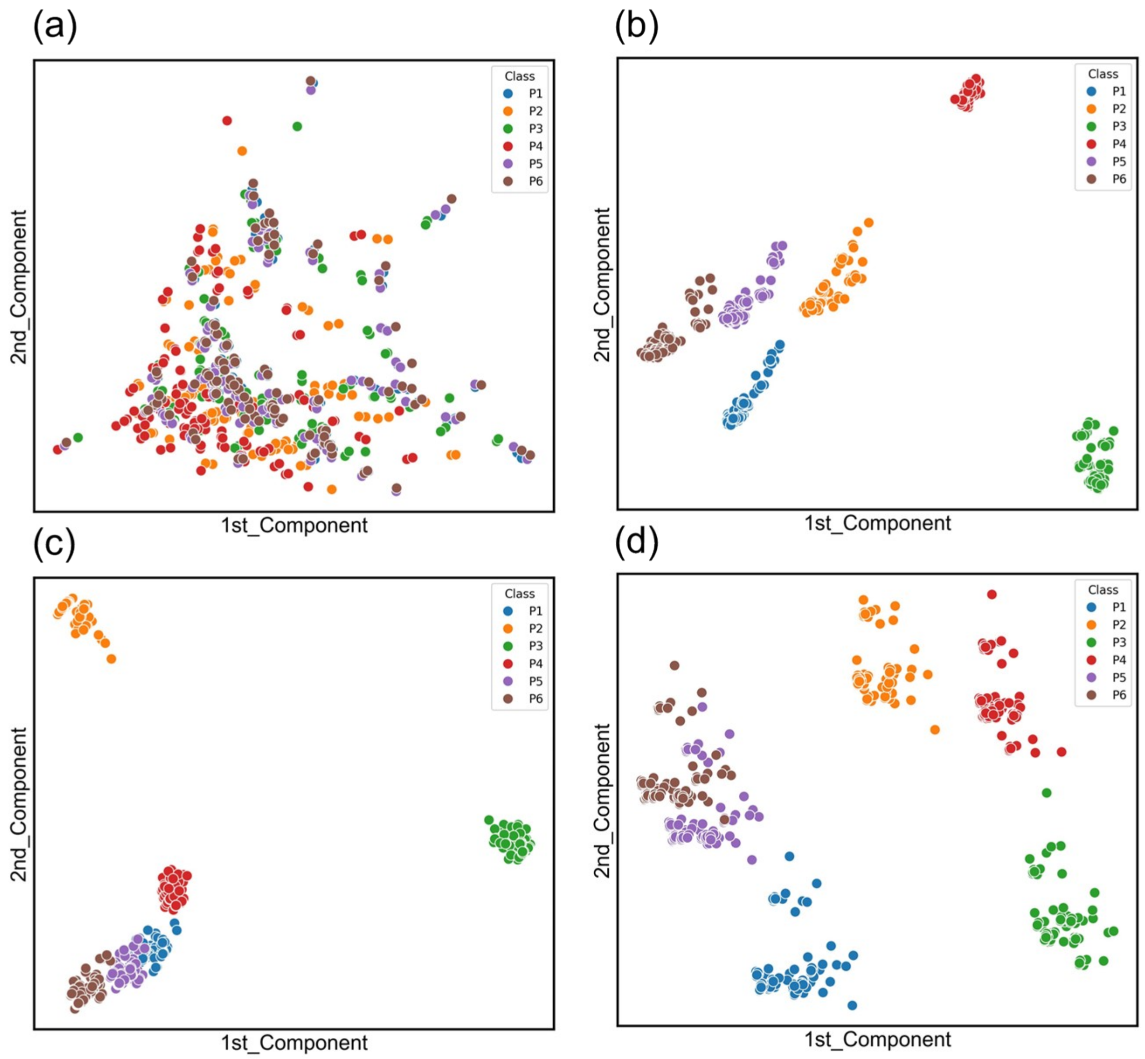

Figure S15. Visualization of the ESIPT molecular dataset based on PCA method for (a) atom-pair, (b) RDKit, (c) ECFP, (d) topotorsion descriptor. Each point represents a molecule. P1, P2, P3, P4, P5 and P6 mean the derivatives of HBO, HBQ, 3HF, HAQ, HBI, and HBT, respectively. The HBO, HBQ, 3HF, HAQ, HBI and HBT derivatives are marked with different colors respectively.

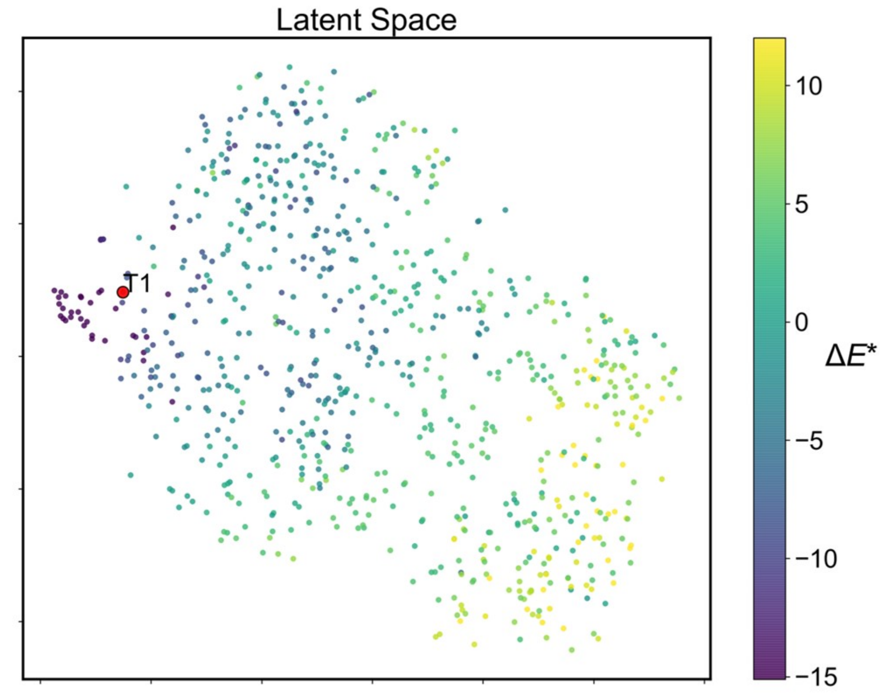

Figure S16. Chemical latent space of our constructed ESIPT dataset and documented ESIPT molecules, with target molecule highlighted in red color point.

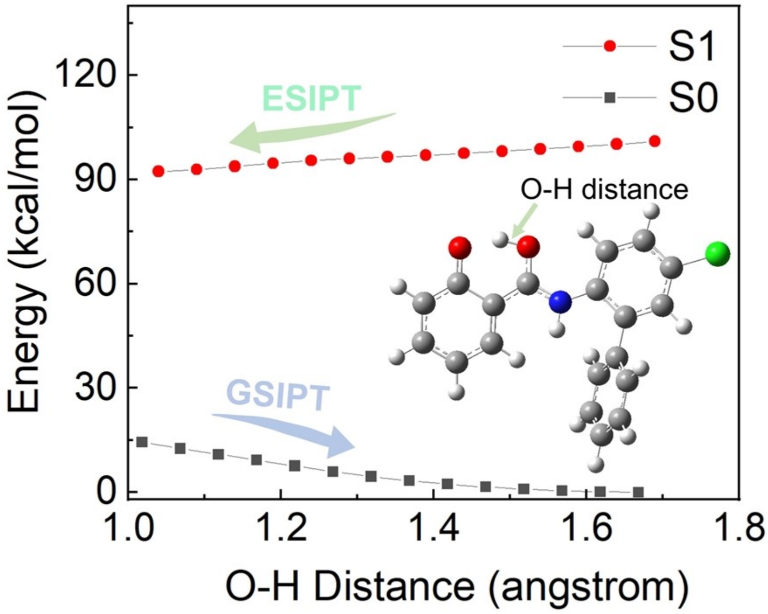

Figure S17. Potential energy curves of the S_0 and S_1 states of **CBHB** along with the H-bond distance in vacuum. The inset shows the stepwise scanned H-bond distance.

S1.5 ADMET Evaluation

While various ADMET properties were predicted by ADMETlab 3.0, safety, pharmacokinetics, logS, and logD are particularly critical for evaluating the applicability of ESIPT molecules as fluorescent probes. In this study, safety was evaluated based on four commonly assessed parameters: AMES toxicity, skin sensitization, hERG inhibition, and the potential for drug-induced liver injury (DILI). A safety score was assigned according to these parameters, with values of +1 for “Excellent,” +0.5 for “Medium,” and +0 for “Poor.” Pharmacokinetic evaluation involved five parameters: VDss, Fu, CL, T1/2, and PPB, using the same scoring system as the safety evaluation. LogD and LogS, which describe the distribution of molecules between lipid and aqueous phases, are crucial factors influencing molecular solubility, bioavailability, and potential applications in physiological environments. The full list of corresponding properties for generated ESIPT molecules can be found in the Supporting Information xlsx file 4.

S2. Δ*E** Prediction Website

A web-based platform using JSME[7] was developed for Δ*E** prediction, where users can either upload their own ESIPT molecules or draw them directly on the website. Note: For optimal prediction accuracy, it is recommended that the core scaffold of the uploaded or drawn molecules aligns with one of the six representative backbones included in the ESIPT dataset—namely HBO, HBQ, HBI, 3HF, HBT, or HAQ. The website address is https://www.aiesipt.asia/. The website page is displayed as follows.

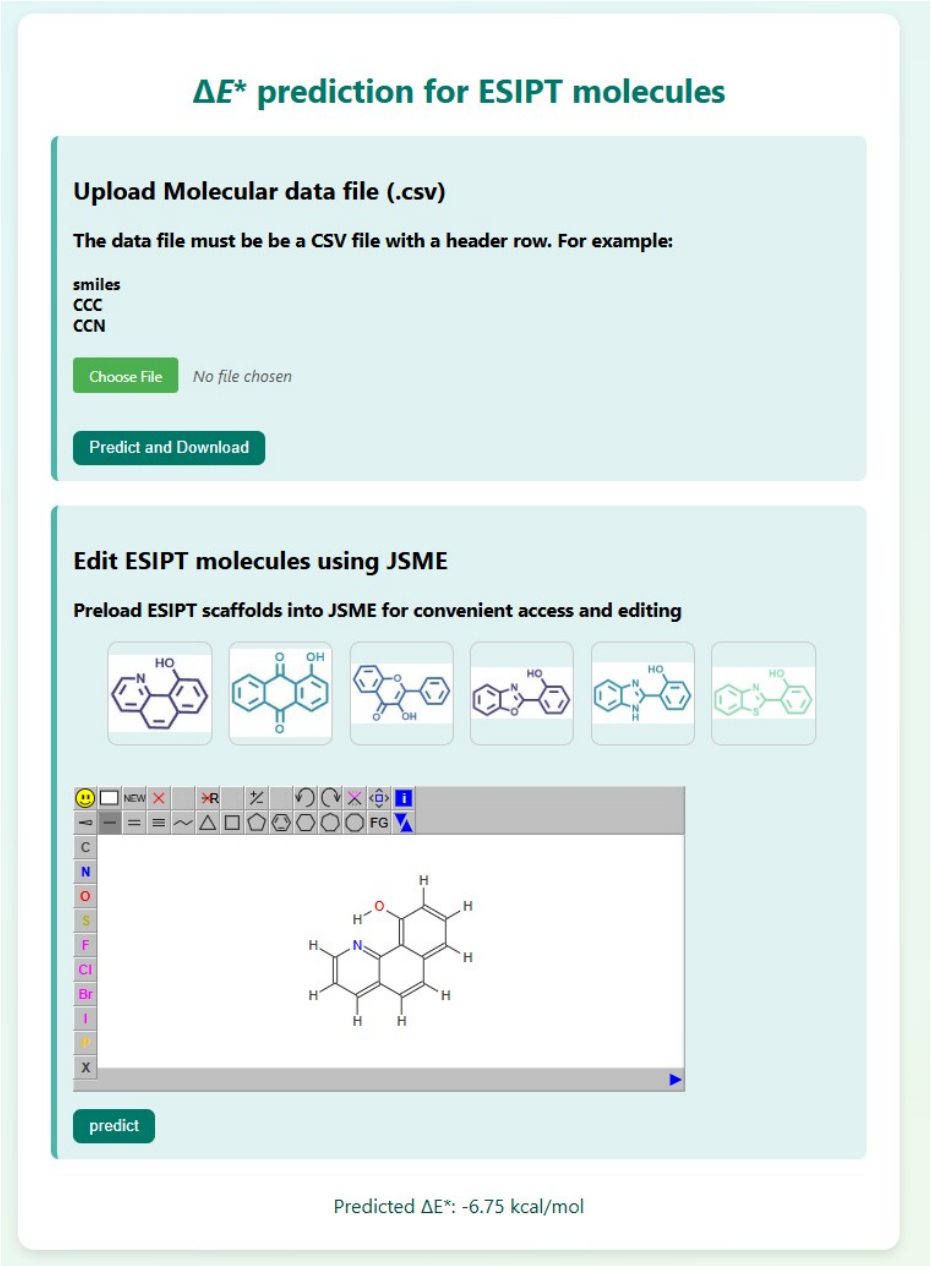

S3. Molecular Generation from NPVAE

NPVAE consists of three components: preprocessing, Encoder, and Decoder. In preprocessing, the compound structure is decomposed into fragments according to certain rules and converted into a corresponding tree structure. In the Encoder, the tree structure obtained from preprocessing and the original compound structure are inputted to calculate the latent variable z. In the Decoder, taking the latent variable z as input, a tree structure is generated using a depth-first algorithm, and then converted back into the corresponding compound structure. Novel ESIPT molecules were generated from the vicinity of T1 molecules with the search radius as 2 in the latent space. The SA scores of generated molecules are evaluated in ADMETlab 3.0. Ultimately, approximately 183 ESIPT compounds were generated. The experiment of NPVAE was performed on a single A100 GPU at the Supercomputing Center of Beijing University of Posts and Telecommunications. We selected T1 as the anchor molecule based on the following considerations: (1) its structural simplicity and synthetic accessibility, as ensuring that AI-designed molecules can be experimentally validated is our primary concern; and (2) the fact that T1 serves as a simple minimal ESIPT probe, which guarantees its applicability.

To further illustrate NPVAE's capability, we additionally performed molecular generation using four different scaffold molecules as anchors, including T1 in the main text (Figure S22). As shown in the figure below, starting from these four target molecules, the model successfully generated a total of 1,260 ESIPT compounds (see Supplementary Files, XLSX file 5). With only four target molecules, the model is able to produce a large number of valid ESIPT structures, demonstrating that AI-based molecular generation is considerably more efficient than manual molecular design.

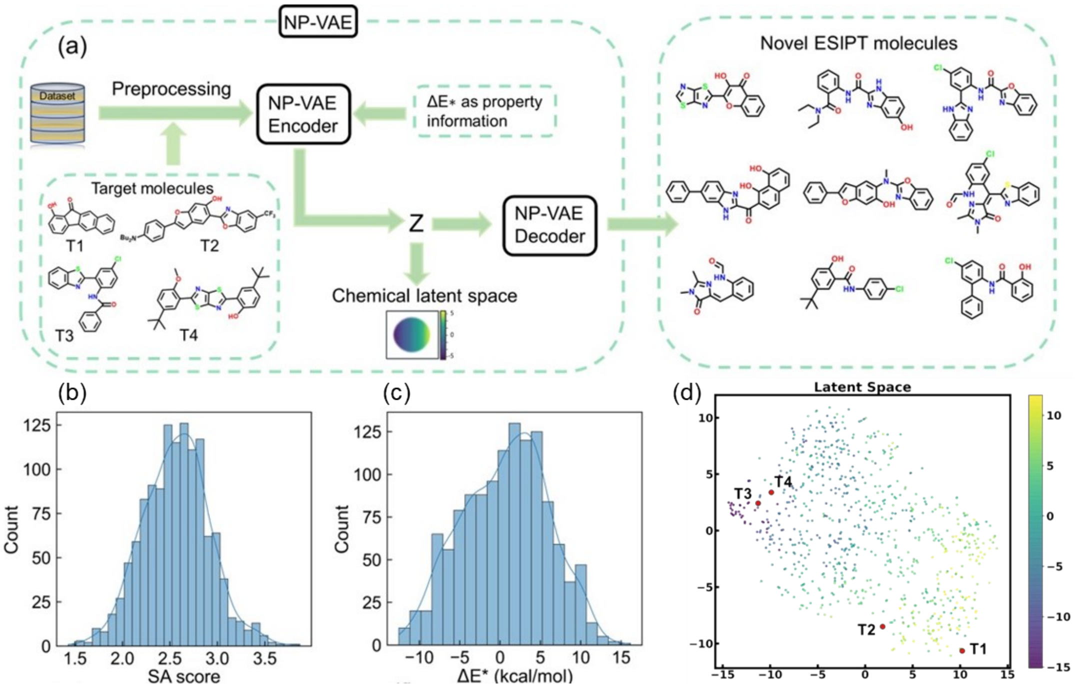

Figure S22. (a) The workflow of NPVAE for ESIPT molecular generation. Distribution of the (b) SA scores and (c) ΔE^* for generated ESIPT molecules. (d) Chemical latent space of generated molecules, with four target ESIPT molecules highlighted in red color.

The generation of structurally novel ESIPT molecules with ΔE^* values comparable to those of target compounds constitutes one of the central objectives of this study. To this end, four target molecules were deliberately selected whose parent scaffolds lie outside the scaffold distribution of the NPVAE training set (Figure S23), inherently resulting in low scaffold similarity between the training data and the target molecules (Figure S24). Consequently, the relatively low structural similarity observed between the generated molecules within the training-set distribution and the target molecules is anticipated. In contrast, selecting target molecules with scaffolds more closely aligned with those represented in the training set would be expected to yield a significantly higher proportion of generated molecules sharing similar scaffolds.

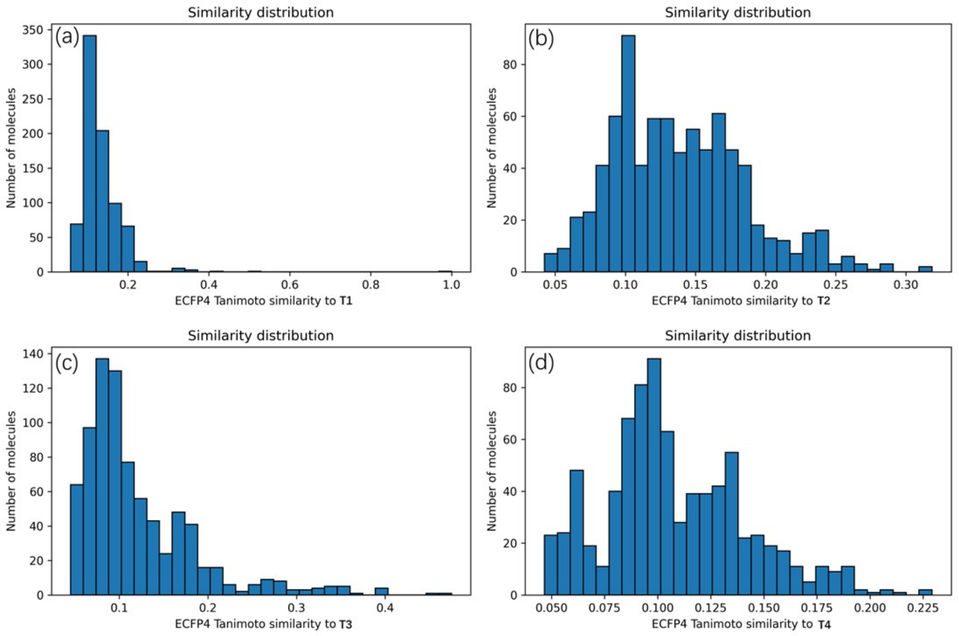

Figure S23. Similarity between the NPVAE training set and the four target molecules.

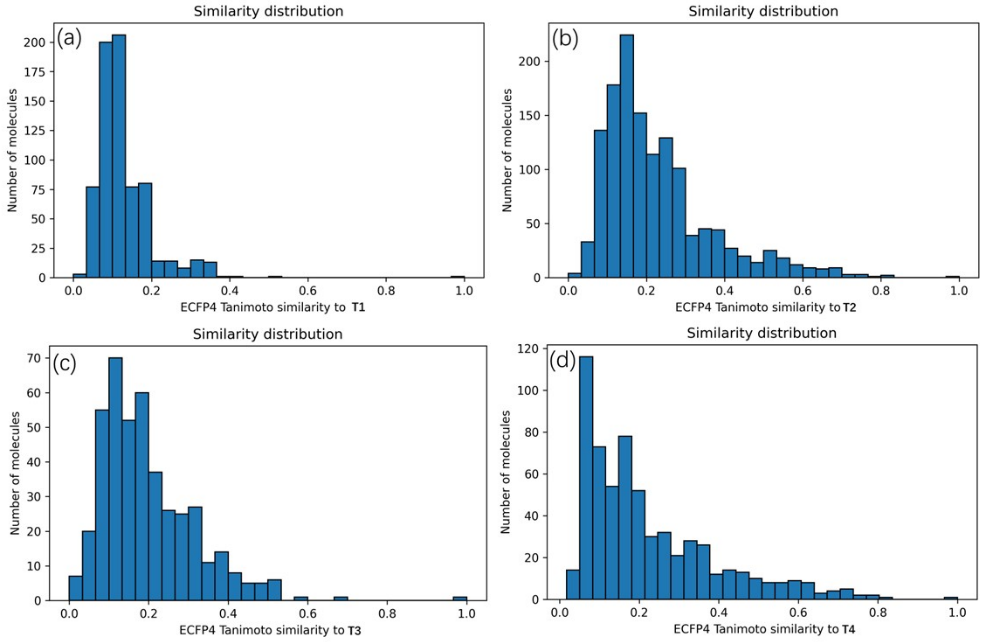

Figure S24. Similarity between the generated molecules and their corresponding target molecules

The *search_radius* parameter defines the search space centered on the target molecule, with *search_radius* = 1 being the default value in the original NPVAE model. In the original NPVAE paper, the training set comprised approximately 76,000 compounds, such that using *search_radius* = 1 was sufficient to generate a large number of candidate molecules for virtual

screening. In contrast, our dataset contains only about 704 molecules. When using *search_radius* = 1 with T1 as the target molecule, only around ten ESIPT molecules were generated, which is insufficient for subsequent virtual screening and synthesis. Therefore, we adopted *search_radius* = 2, which yielded 184 generated molecules. However, increasing *search_radius* expands the search space farther from the reference molecule, inevitably leading to a fraction of generated molecules that deviate more substantially from the reference in both structure and properties.

Another factor contributing to the structural diversity is the model's loss function. In the original NPVAE paper (the following content is excerpted from *Variational autoencoder-based chemical latent space for large molecular structures with 3D complexity*), the loss function is defined as follows:

$$L = \alpha \cdot CE\left(y_r, u_{L_r}\right) + \beta \cdot \sum_i CE\left(y_{\tau,i}, u_\tau\right) + \gamma \cdot \sum_j CE\left(y_{s,j}, u_{L_s}\right) + \delta \cdot \sum_j CE\left(y_{b,j}, u \text{[illegible]}\right.$$
$$\left(y_p, u_{L_p}\right) + \zeta \cdot D_{KL}[Q(z|X)||P(z)]$$

As can be seen, in addition to the first five reconstruction loss terms commonly used in standard VAE models (which aim to ensure high structural similarity between the generated molecules and the reference molecules) and the conventional latent-space regularization term, the loss function also includes an additional property-prediction loss term: MSE((y_p, u_{Lp})). This term makes a non-negligible contribution to the total loss. In our training process, Δ*E** is the target functional property incorporated into this term. By embedding property information into the latent space, the model endows the latent space with a property gradient, such that molecules closer to the target molecule in latent space are expected to exhibit properties (Δ*E** in this work) more similar to those of the target molecule.

To verify that the generated molecules are also sensitive to Δ*E**, we employed our Δ*E** prediction model to estimate the Δ*E** values of the generated molecules and compared them with those of the corresponding target molecules (Figure R4). For T2 and T3, both the predicted and calculated Δ*E** values of the target molecules are located near the center of the Δ*E** distributions of their generated counterparts, indicating that the generation process is sensitive

to Δ*E**. This observation also suggests that the prediction model is relatively valid for T2 and T3 as well as for their corresponding generated molecules. In contrast, the Δ*E** values of T1 and T4 do not fall near the centers of the Δ*E** distributions of their generated molecules. This discrepancy arises because the prediction model is not well applicable to T1 and T4 and their corresponding generated molecules. Specifically, the calculated and predicted Δ*E** values for T1 are 10 kcal/mol and −6.4 kcal/mol, respectively, while those for T4 are 3.2 kcal/mol and −7.5 kcal/mol. Consequently, the predicted Δ*E** values of molecules generated based on T1 and T4 are also inaccurate. Importantly, this limitation reflects the restricted applicability domain of the prediction model rather than a failure of the generative model itself.

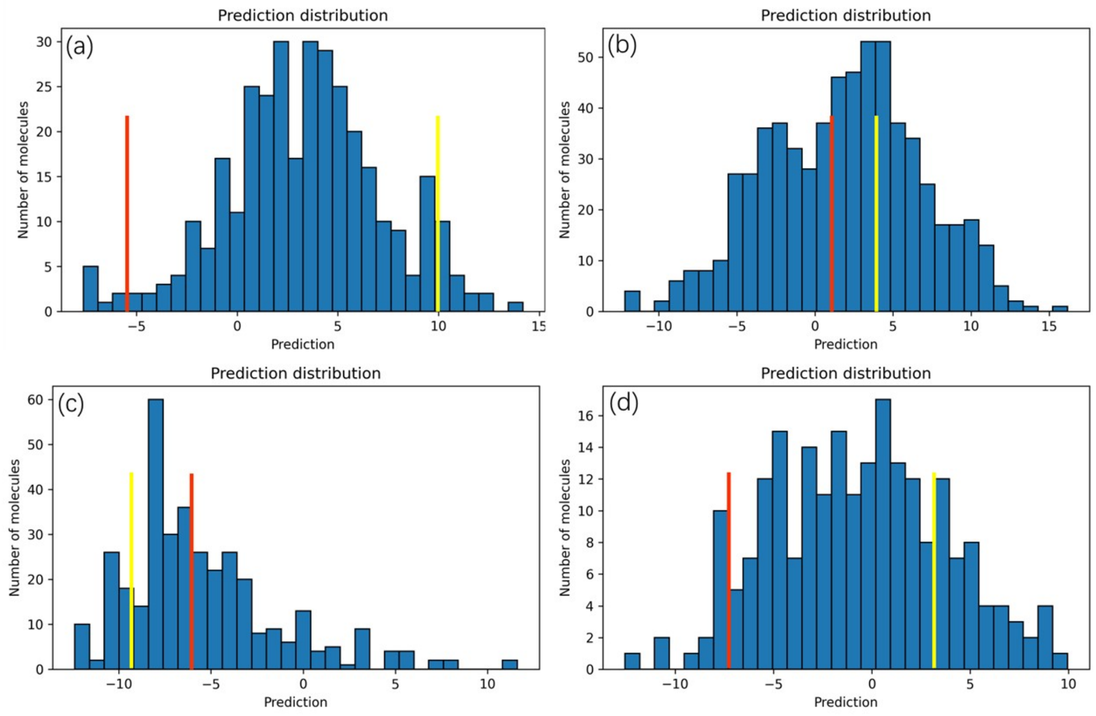

Figure S25. Distributions of the predicted Δ*E** values for molecules generated from T1 (a), T2 (b), T3 (c), and T4 (d). The red and yellow vertical bars in the figure represent the predicted and DFT-calculated values of the target molecules, respectively.

Finally, we would like to note that Δ*E** in ESIPT is influenced by many factors. Empirically, structural similarity between two ESIPT molecules often correlates with similar Δ*E** values, but this relationship is not deterministic. Accordingly, the presence of a subset of generated molecules that differ substantially in structure from the reference molecules is permissible from both the NPVAE training and molecular generation principles, as well as

from a chemical perspective.

S4. Materials and Instruments

Materials. All the chemicals including solvents, reagents, and catalysts were purchased from Sigma-Aldrich, Alfa Aesar chemical company, Sunatech Co., Ltd. Unless otherwise specified, such chemicals were used without any further purification.

General characterization. NMR spectra were recorded on a Bruker ARX 400 NMR spectrometer. Chemical shifts are recorded in parts per million referenced according to residual solvent ($CDCl_3$ = 7.26 ppm) in ^{1}H NMR and ($CDCl_3$ = 77.0 ppm) in ^{13}C NMR.

Scheme S1. Five ESIPT molecules generated by NPVAE.

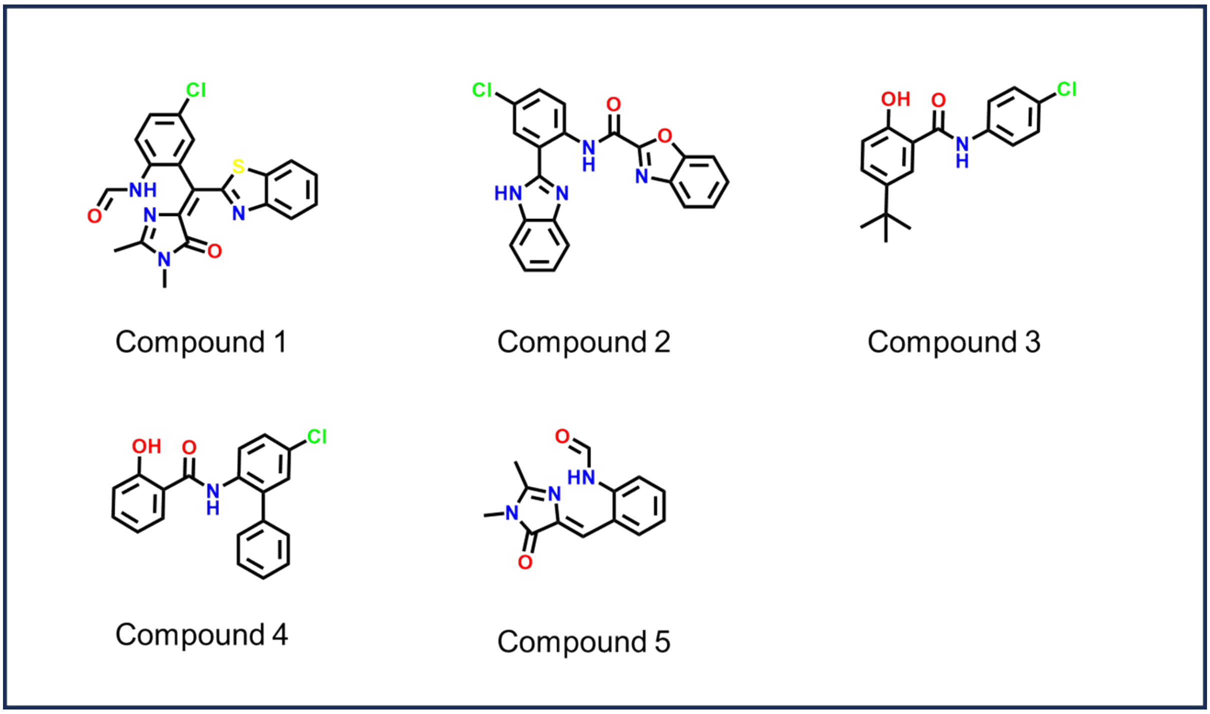

Corresponding properties of five AI-generated ESIPT molecules.

Compounds	SA score	Pharmacokinetics score	Safety score	Total score	Predicted ΔE^* (kcal/mol)
1	3.098	2	1	3	-0.57
2	2.278	2	1.5	3.5	-7.67
3	1.752	1.5	1.5	3	-4.27
4	1.675	2	1	3	-7.45
5	2.861	4	1	5	-4.08

[a] The total score is defined as the sum of the pharmacokinetics score and the safety score.

Synthesis of Compound 3. In a dry reaction flask, dissolve 5-(1,1-dimethylethyl) salicylic acid **Compound 3i** (1.00 g, 5.15 mmol, 1.0 equiv.) in 50 mL of anhydrous dichloromethane. Add 1,3-dicyclohexylcarbodiimide **DCC** (1.64 g, 7.96 mmol, 1.1 equiv.) to activate the carboxylic acid, stirring until the intermediate activated ester is formed. Sequentially introduce 4-Chloroaniline **Compound 3ii** (0.66 g, 5.15 mmol, 1.0 equiv.) and 4-dimethylaminopyridine **DMAP** (88 mg, 0.72 mmol, 0.1 equiv.) into the reaction mixture. Stir the mixture at room temperature for 24 hours. Upon completion, the solvent was removed under reduced pressure, and the mixture was extracted with deionized water and DCM. The crude product was separated by column chromatography using PE:DCM (2/1, v/v) as the eluent to give the desired **Compound 8** (1.30 g, 83%) as a white product. ^{1}H NMR (400 MHz, Chloroform-d): δ 11.64 (s, 1H), 7.89 (s, 1H), 7.59–7.50 (m, 3H), 7.42–7.35 (m, 3H), 6.99 (d, J = 8.7 Hz, 1H), 1.35 (s, 9H). ^{13}C NMR (101 MHz, Chloroform-d): δ 168.75, 159.60, 135.40, 132.71, 130.62, 129.33, 122.82, 121.47, 118.72, 113.77, 34.36, 31.54.

Synthesis of Compound 4. In a dry reaction flask, dissolve salicylic acid **Compound 4i** (1.00 g, 7.24 mmol, 1.0 equiv.) in 50 mL of anhydrous dichloromethane. Add 1,3-dicyclohexylcarbodiimide **DCC** (1.64 g, 7.96 mmol, 1.1 equiv.) to activate the carboxylic acid,

stirring until the intermediate activated ester is formed. Sequentially introduce 5-chloro-[1,1'-biphenyl]-2-amine **Compound 4ii** (1.47 g, 7.24 mmol, 1.0 equiv.) and 4-dimethylaminopyridine **DMAP** (88 mg, 0.72 mmol, 0.1 equiv.) into the reaction mixture. Stir the mixture at room temperature for 24 hours. Upon completion, the solvent was removed under reduced pressure, and the mixture was extracted with deionized water and DCM. The crude product was purified by column chromatography using PE/DCM (2/1, v/v) as the eluent, followed by recrystallization from DCM/PE to give the desired **Compound 4** (2.04 g, 87%) as a white needle-like solid. ^{1}H NMR (400 MHz, Chloroform-d): δ 11.86 (s, 1H), 8.36 (d, J = 8.8 Hz, 1H), 8.06 (s, 1H), 7.53 (dt, J = 13.2, 6.8 Hz, 3H), 7.44–7.35 (m, 4H), 7.33 (s, 1H), 7.00 (s, 1H), 6.85 (d, J = 6.4 Hz, 1H), 6.73 (t, J = 8.2 Hz, 1H). ^{13}C NMR (101 MHz, Chloroform-d): δ 167.75, 161.79, 136.23, 134.51, 134.24, 132.30, 129.92, 129.72, 129.32, 128.98, 128.72, 128.28, 124.75, 122.76, 118.82, 114.18.

Scheme S2. Synthetic route of five generated ESIPT molecules.

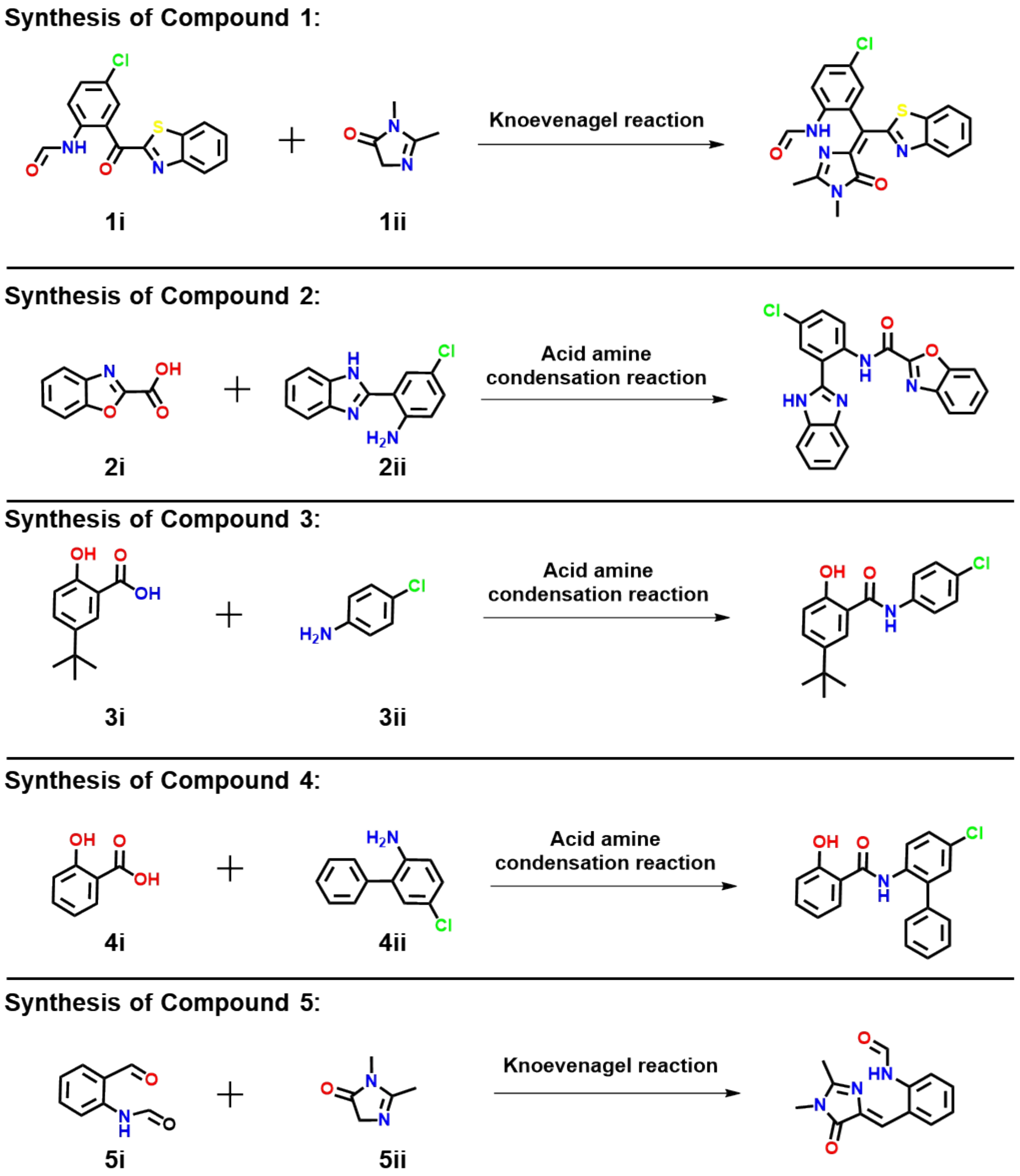

Table S2. The absorption and fluorescence properties of **TCHB** and **CBHB** in different solvents.

Compounds		CHX	TOL	DCM	ACN	MeOH
**TCHB**	λ_{abs} (nm)	318, 268	320, 288	318, 268	316, 269	312, 269
	λ_{Flu} N* (nm)	372,	357	393	430	438
	λ_{Flu} T* (nm)	485	495	495	500	
**CBHB**	λ_{abs} (nm)	315	315	315	302	305, 276
	λ_{Flu} N* (nm)	355	355	395	425	435
	λ_{Flu} T* (nm)	469	473	467	490	

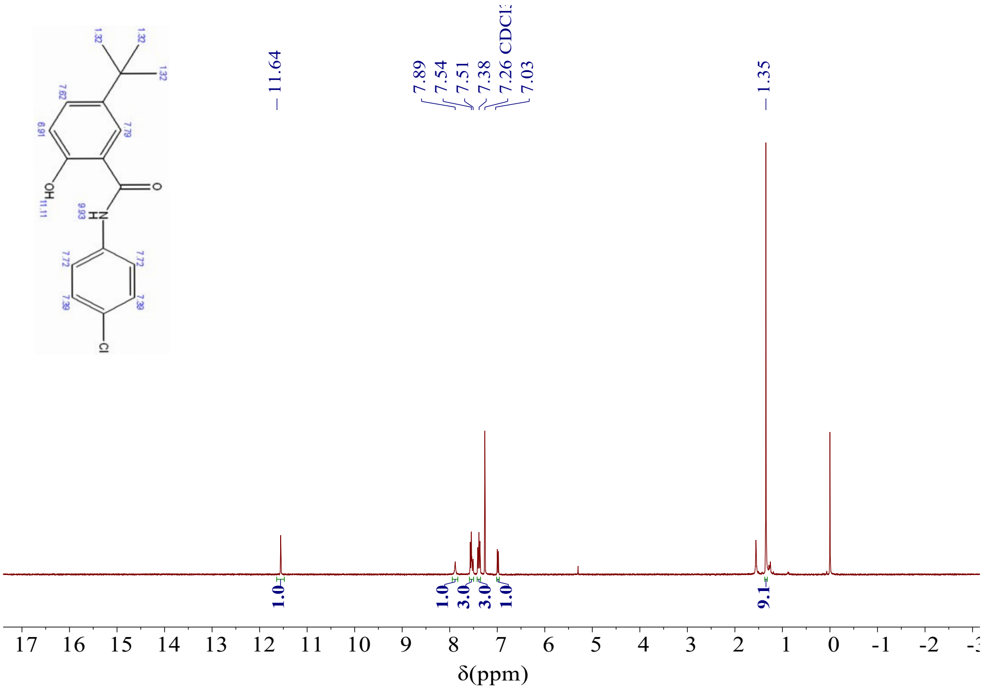

Figure S26. ^{1}H NMR spectrum of **Compound 3 (TCHB)** in $CDCl_3$.

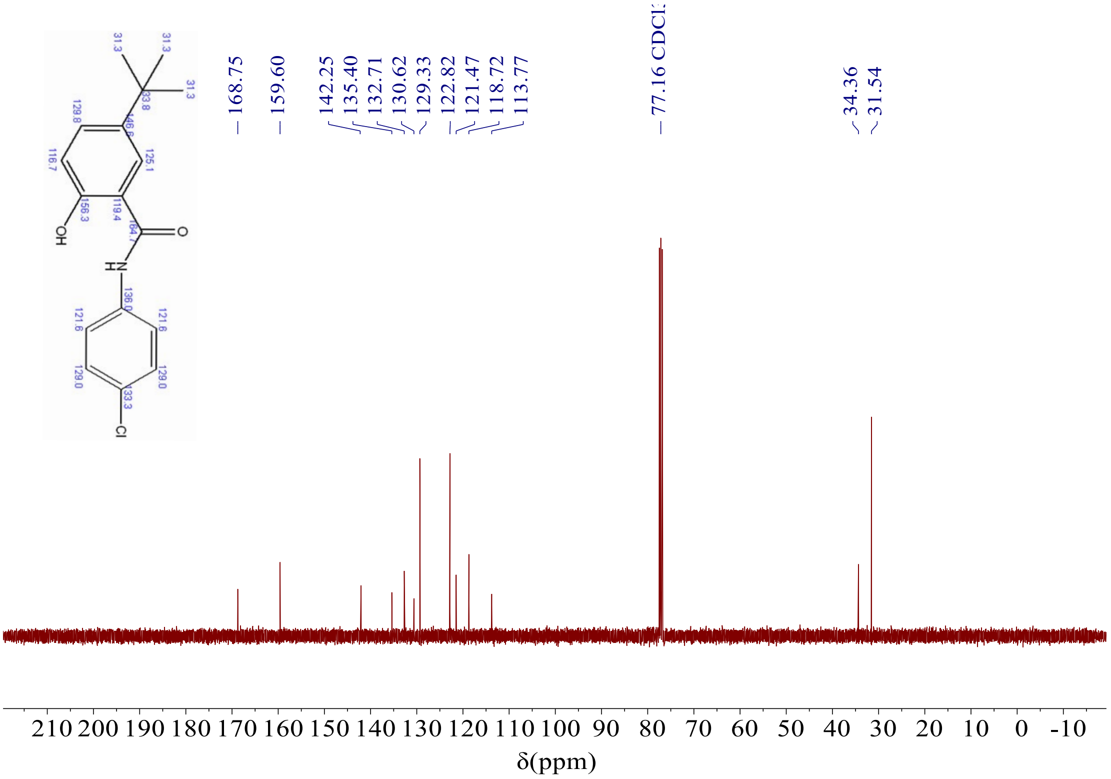

Figure S27. ^{13}C NMR spectrum of **Compound 3 (TCHB)** in $CDCl_3$.

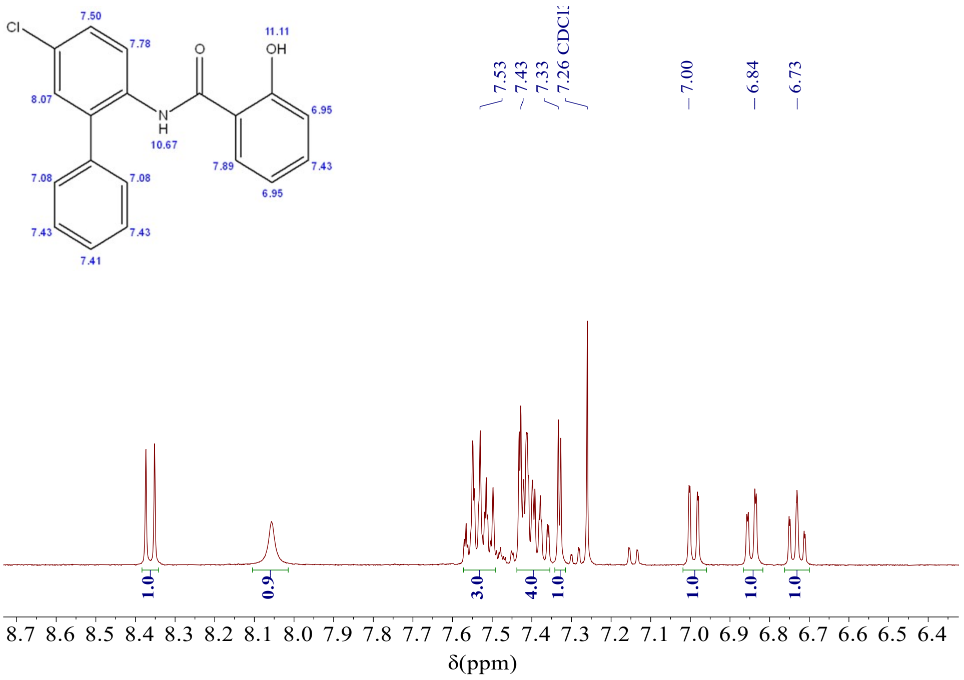

Figure S28. ^{1}H NMR spectrum of **Compound 4 (CBHB)** in $CDCl_3$.

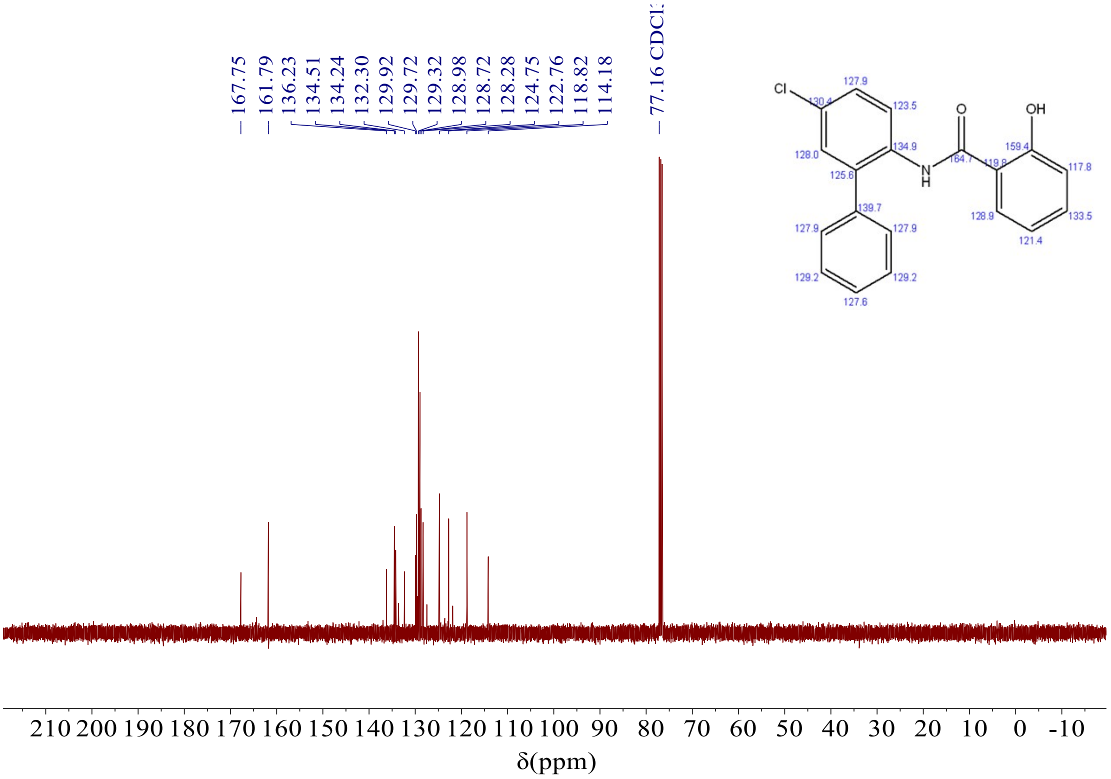

Figure S29. ^{13}C NMR spectrum of **Compound 4 (CBHB)** in $CDCl_3$.

S5. References

1 M. J. Frisch, G. W. Trucks, H. B. Schlegel, G. E. Scuseria, M. A. Robb, J. R. Cheeseman, G. Scalmani, V. Barone, G. A. Petersson, H. Nakatsuji, X. Li, M. Caricato, A. V. Marenich, J. Bloino, B. G. Janesko, R. Gomperts, B. Mennucci, H. P. Hratchian, J. V. Ortiz, A. F. Izmaylov, J. L. Sonnenberg, Williams, F. Ding, F. Lipparini, F. Egidi, J. Goings, B. Peng, A. Petrone, T. Henderson, D. Ranasinghe, V. G. Zakrzewski, J. Gao, N. Rega, G. Zheng, W. Liang, M. Hada, M. Ehara, K. Toyota, R. Fukuda, J. Hasegawa, M. Ishida, T. Nakajima, Y. Honda, O. Kitao, H. Nakai, T. Vreven, K. Throssell, J. A. Montgomery Jr., J. E. Peralta, F. Ogliaro, M. J. Bearpark, J. J. Heyd, E. N. Brothers, K. N. Kudin, V. N. Staroverov, T. A. Keith, R. Kobayashi, J. Normand, K. Raghavachari, A. P. Rendell, J. C. Burant, S. S. Iyengar, J. Tomasi, M. Cossi, J. M. Millam, M. Klene, C. Adamo, R. Cammi, J. W. Ochterski, R. L. Martin, K. Morokuma, O. Farkas, J. B. Foresman and D. J. Fox, Gaussian 16 Rev. A.03., 2016.

2 F. Pedregosa, G. Varoquaux, A. Gramfort, V. Michel, B. Thirion, O. Grisel, M. Blondel, P. Prettenhofer, R. Weiss, V. Dubourg, J. Vanderplas, A. Passos, D. Cournapeau, M. Brucher, M. Perrot and É. Duchesnay, Scikit-learn: Machine Learning in Python, *J. Mach. Learn. Res.*, 2011, **12**, 2825–2830.

3 M. Abadi, P. Barham, J. Chen, Z. Chen, A. Davis, J. Dean, M. Devin, S. Ghemawat, G. Irving, M. Isard, M. Kudlur, J. Levenberg, R. Monga, S. Moore, D. G. Murray, B. Steiner, P. Tucker, V. Vasudevan, P. Warden, M. Wicke, Y. Yu and X. Zheng, 12th USENIX Symposium on Operating Systems Design and Implementation (OSDI 16), 2016.

4 R. Nilakantan, N. Bauman, J. S. Dixon and R. Venkataraghavan, Topological torsion: a new molecular descriptor for SAR applications. Comparison with other descriptors, *J. Chem. Inf. Comput. Sci.*, 1987, **27**, 82-85.

5 J. L. Durant, B. A. Leland, D. R. Henry and J. G. Nourse, Reoptimization of MDL Keys for Use in Drug Discovery, *J. Chem. Inf. Comput. Sci.*, 2002, **42**, 1273-1280.

6 K. Yang, K. Swanson, W. Jin, C. Coley, P. Eiden, H. Gao, A. Guzman-Perez, T. Hopper, B. Kelley, M. Mathea, A. Palmer, V. Settels, T. Jaakkola, K. Jensen and R. Barzilay, Analyzing Learned Molecular Representations for Property Prediction, *J. Chem. Inf. Model.*, 2019, **59**, 3370-3388.

7 B. Bienfait and P. Ertl, JSME: a free molecule editor in JavaScript, *J. Cheminform.*, 2013, **5**, 24.
